# Immediate effects of diacutaneous fibrolysis in athletes with hamstring shortening. A randomized within-participant clinical trial

**DOI:** 10.1371/journal.pone.0270218

**Published:** 2022-07-05

**Authors:** Aïda Cadellans-Arróniz, Carlos López-de-Celis, Jacobo Rodríguez-Sanz, Albert Pérez-Bellmunt, Noé Labata-Lezáun, Vanessa González-Rueda, Luis Llurda-Almuzara, Pere Ramón Rodríguez-Rubio

**Affiliations:** 1 Faculty of Medicine and Health Sciences, Universitat Internacional de Catalunya, Carrer de Josep Trueta, Barcelona, Spain; 2 ACTIUM Functional Anatomy Group, Carrer de Josep Trueta, Barcelona, Spain; 3 Fundació Institut Universitari per a la recerca a l’Atenció Primaria de Salut Jordi Gol i Gurina, Barcelona, Spain; Prince Sattam Bin Abdulaziz University, College of Applied Medical Sciences, SAUDI ARABIA

## Abstract

**Background:**

Diacutaneous fibrolysis is a non-invasive instrumental physiotherapeutic technique, used to treat mechanical or inflammatory pain and normalize function in the musculoskeletal system. Different studies have reported positive effects on range of motion, strength or function in musculoskeletal disorders, mainly in the upper extremity. The incidence and recurrence rates of hamstring injuries are high in many sports. However, there are no studies assessing these parameters in the hamstring and gluteus maximus in athletes. Objective. To evaluate the immediate and 30 minutes post-treatment effects of a single diacutaneous fibrolysis session on hamstring length, flexibility, muscle strength, myoelectrical activity and lower limb performance in athletes with hamstring shortening.

**Methods:**

A randomized within-participant clinical trial. Sixty-six athletes with hamstring shortening were recruited. A single session of diacutaneous fibrolysis was applied following the cetripetal protocol to the gluteus maximus, biceps femoris and semitendinosus of for the experimental lower limb, whereas the control limb was not treated. Hamstring length (Passive knee extension test), hamstring and low back flexibility (Modified back saver sit and reach test), hamstring and gluteus maximus strength and electrical activity (dynamometry and surface electromyography, respectively) and lower limb performance (Countermovement Jump) were tested before treatment (T0), after treatment (T1), and 30 minutes post-treatment (T2).

**Results:**

We only found a statistically significant difference between T0-T2 for the hamstring length favouring the experimental limbs (p<0.001). There were no statistically significant changes for hamstring and lower back flexibility, strength and electrical activity outcomes between groups. In the countermovement jump, we found a decrease of 0.58 cm in the high jump and a decrease of 9.19 N in the power jump at T1. These values recovered and improved at T2. However, these changes were not statistically significant (p>0.05).

**Conclusions:**

A single session of diacutaneous fibrolysis in athletes with hamstring shortening, following the centripetal protocol for the posterior part of the thigh, produces improvements in hamstring length 30 minutes after, and in gluteus maximus strength immediately and 30 minutes after the treatment. It seems to have no effects on the overall hamstring and lower back flexibility or myoelectric activity. Importantly, the lower limb performance was not impaired after the treatment.

## 1. Introduction

Diacutaneous fibrolysis (DF) is a non-invasive instrumental physiotherapeutic technique developed following cyriax deep friction massage principles [1]. It is used to treat mechanical or inflammatory pain and normalize function in the musculoskeletal system [2]. This technique uses metal hooks that seem to allow a deeper and more precise application than the manual approach. It is characterized by cetriptal application, involving the treatment of surrounding tissues related to the target musculature [3, 4]. Different DF studies have reported improvements in range of motion (ROM) for musculoskeletal disorders such as subacromial impingement syndrome [5], painful shoulder [6] or temporomandibular disorders [7]. After DF, greater strength and function has also been reported [4, 8]. It is hypothesized that after DF, normal muscle mechanics could be recovered to improve muscle performance [8]. This statement is supported by the improvements in muscle plasticity, sarcomerogenesis and extracellular matrix remodelling observed after the application of myofascial release techniques [9]. In addition, previous studies also show significant muscle strength and excitability improvements in the gastrocnemius after being treated with DF, compared to a sham DF group, as well as a higher neuromuscular efficiency during explosive force production [8]. However, no studies have been found that evaluate muscle flexibility changes that could explain the ROM improvements.

The relationship between hamstring flexibility and injury has been documented. More specifically, the lack of hamstring flexibility has been pointed out as a risk factor for hamstring strains and load alterations in the biomechanics of the lower limbs [10, 11]. Specifically, it has been described that the landing after vertical jumping would cause hamstring injuries when athletes suffer muscular stiffness conditions [12]. Hamstring shortening is further characterized by altered muscle recruitment patterns, as well as decreased strength [13]. It has been shown that, when hamstring flexibility is improved, the maximum concentric and eccentric strength are also increased [14]. And, in this sense, improvements in muscle strength are recommended to prepare the athletes to sustain the high work rates throughout training and matches and mitigate their injury risk [15].

The incidence and recurrence rates of hamstring injury are high in many sports [16]. It is estimated that almost 30% of lower limb injuries are attributable to the hamstrings [17]. These injuries range from mild alterations to complete loss of fiber organization, being the hamstring strain injury the most common one [11]. In professional athletes, a prolonged recovery time and a higher reinjury rate has been associated with an involvement of the intramuscular tendon [18]. Within the hamstrings, the biceps femoris has the highest injury rate (84%) followed by semimembranosus (12%) and the semitendinosus (4%) [13]. Specifically, the biceps femoris has shown 54% risk of re-injury, with 86% of subsequent injuries of equal or greater severity [18]. It is described that muscular stiffness and overload may alter the performance during sports activities, causing pain or affecting the normal muscle mechanics, and thus impairing strength and motor coordination [13].

The use of soft-tissue release techniques seems to prevent those dysfunctions without strength impairment [9, 19]. Different soft tissue mobilizations techniques have been studied before in order to evaluate athletic performance effects in athletes [12, 14, 15]. It has also been reported that manual techniques could increase blood flow and temperature but also normalize muscle activity. DF is widely used in sports for therapeutic and preventive purposes. We have only found one DF study on anterior knee pain in athletes, but changes in muscular strength and performance were not assessed. To the best of our knowledge, no study evaluated DF effects in athletes with hamstring shortening on myoelectric activity while performing a vertical countermovement jump, a test commonly used to monitor strength performance and conditioning in lower limbs. Thus, this study aims to evaluate the immediate and 30 minutes post-treatment effects of a single DF session on hamstring length and flexibility as well as muscle strength, electrical activity and lower limb performance in athletes with hamstring shortening.

## 2. Methods

### 2.1. Study design

**Fig 1:**
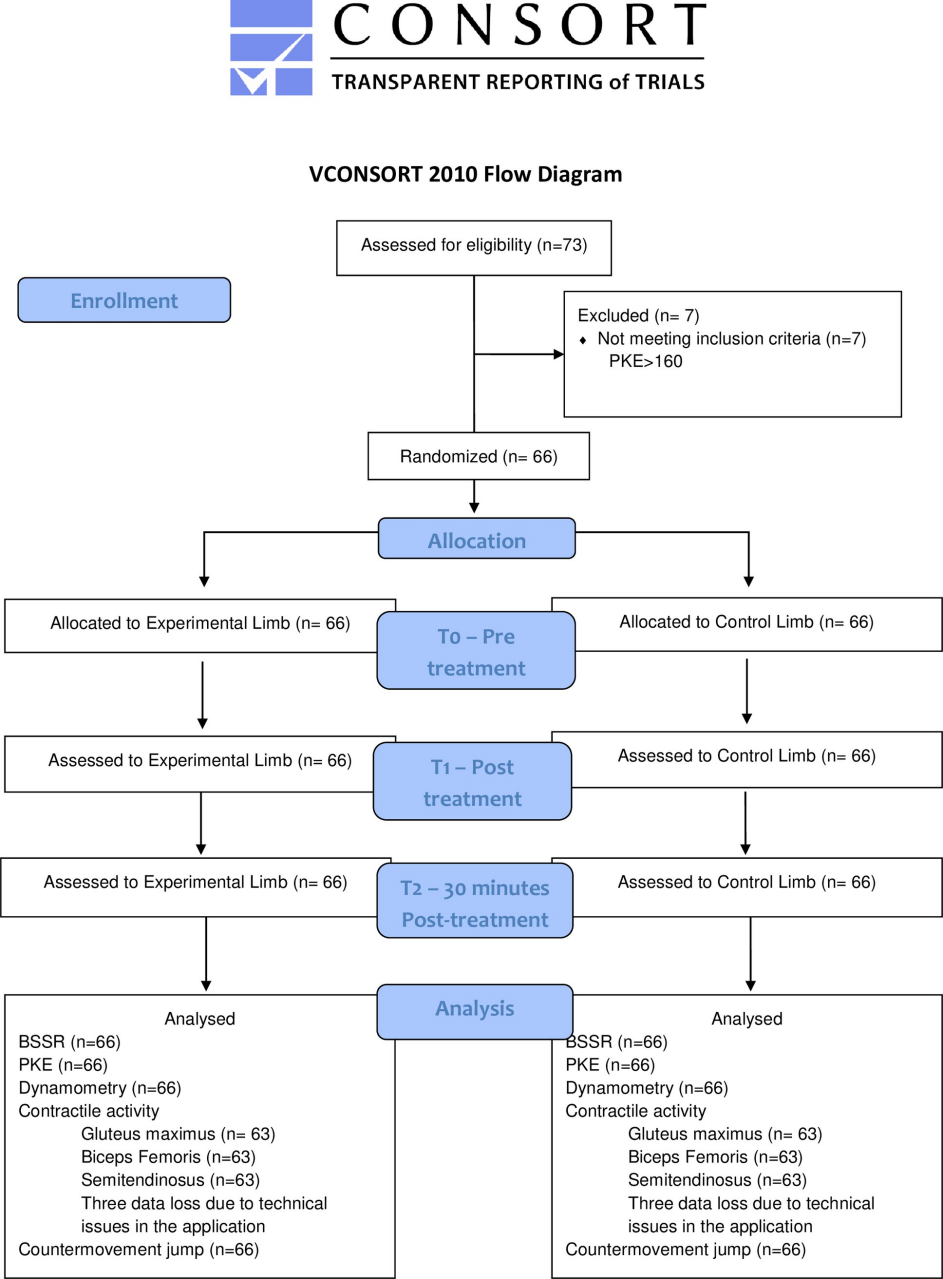
CONSORT flow diagram.

A randomized within-participant, longitudinal, single blinded (evaluator) controlled clinical trial was conducted at the Universitat Internacional de Catalunya research laboratory. The study was registered at www.clinicaltrials.gov (NCT04827082). The local ethics committee of Universitat Internacional de Catalunya –CER (Comitè Ètic de Recerca) approved the study protocol (study Code: FIS-2020-04). The study procedures were conducted following the declaration of Helsinki (World Medical Association, 2013). Informed consent was obtained from all participants. This article is reported following CONSORT 2010 [20] and TIDier guidelines. CONSORT flow diagram is shown in Fig 1

### 2.2. Sample size calculation

The sample size was calculated based on previous pilot work using the GRANMO 7.12 program. A two-sided test analysis (two independent means), assuming an α risk of 0.05 and a β risk of 0.20 (i.e., 80% power) was performed, for an expected improvement of 4.0 cm (SD 7.56 cm) in the Passive knee extension test (PKE). Assuming an estimated dropout rate of 15%, 66 participants (66 intervention limbs, 66 control limbs) were required.

### 2.3. Subjects

Using different dissemination channels of the university, sixty-six athletes from the Faculty of Medicine and Health Sciences of the Universitat Internacional de Catalunya, were recruited from April 1st to May 19^th^ 2021 to voluntarily participate in the study, after signing the informed consent.

The inclusion criteria comprised 1) being athletes over 18 years old, 2) being registered in a sport club or institution where they compete and practice sports on a regular basis and 3) having a hamstring shortening PKE <160° [21]. Exclusion criteria were: any contraindication related to diacutaneous fibrolysis, such us poor skin or trophic condition, taking anticoagulants, suffering from any inflammatory process or recent musculoskeletal lower limb injury (< 6 month).

### 2.4. Randomization and allocation

The randomization was carried out at a limb level, regardless of the laterality. One limb was randomly assigned for DF treatment, and the other limb was considered as control (no intervention). For the randomization process, an external evaluator generated a randomization list before recruiting the athletes with a computer programme (www.random.org) that generated a list of randomized numbers (1 to 66). The evaluator was unaware of the group assignment.

### 2.5. Data collection

Two clinical researchers, who were blind to experimental/control limb assignment, collected and recorded the measurements. All outcomes were measured at baseline (T0), after the DF intervention (T1) and 30 minutes after the DF intervention (T2), on both limbs.

### 2.6. Outcomes

The outcome assessment procedure had an average duration of 10 minutes approximately and was carried out in the order that variables are shown in this section. Hamstring length was considered the primary outcome, whereas flexibility, muscle strength, electrical activity and lower limb performance were the secondary outcomes.

#### 2.6.1. Hamstring length

**Fig 2:**
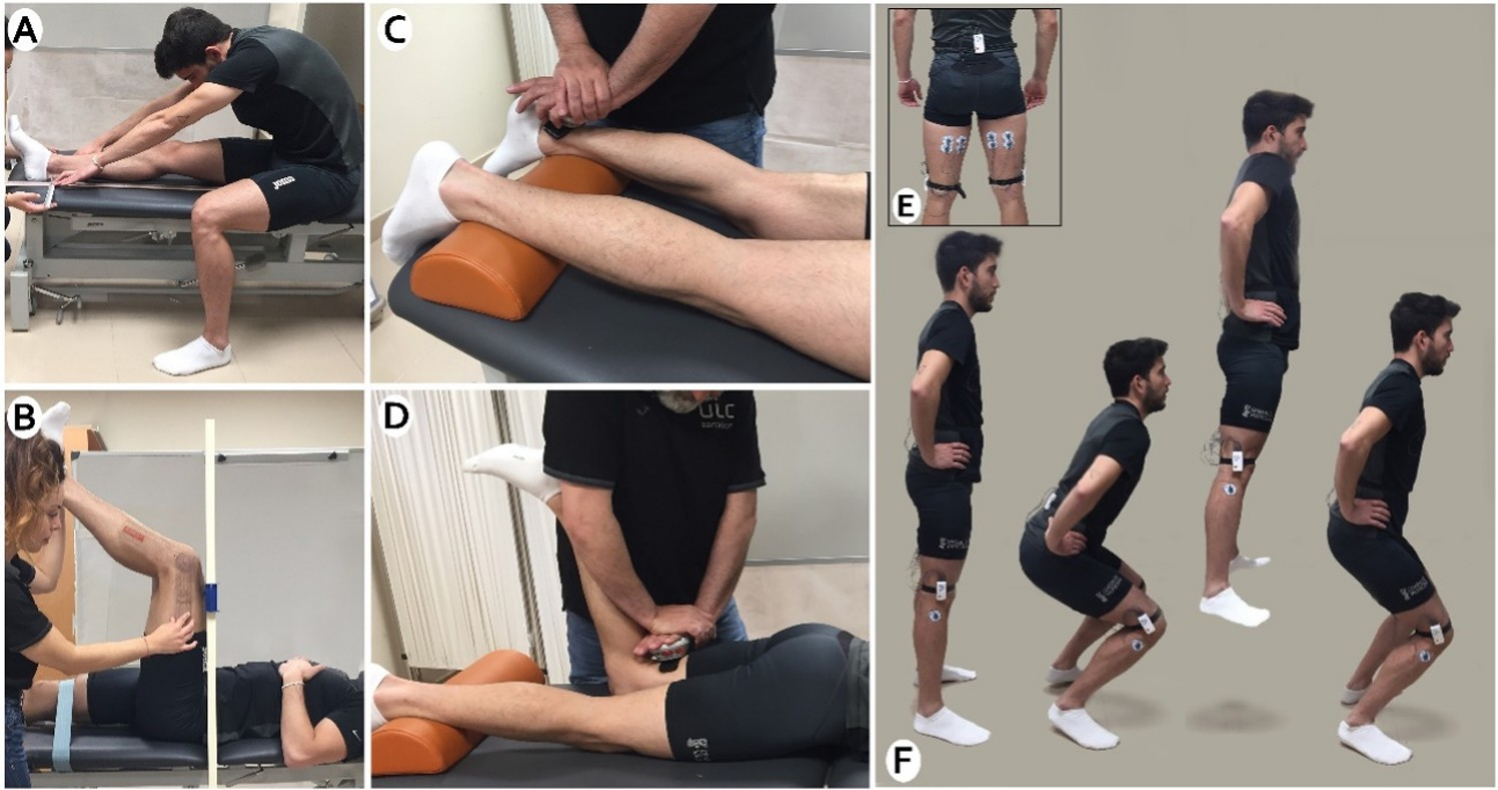
A) Modified Back Saver Sit and reach test. B) Passive Knee Extension Test. C) Hamstring strength. D) Gluteus strength. E) Surface electromyography. F) Countermovement jump.

Hamstring length was assessed using the PKE test. It is based on the measurement of the popliteal angle by means of a universal goniometer and has shown excellent reliability (ICC = 0.96–0.97) in previous studies. The participant was in the supine position with the non-tested leg stabilized, to avoid any compensation of the contralateral hip. A 90° hip flexion was performed on the tested leg and, from this position, the knee was passively extended to the point of firm resistance to movement. In order to ensure the initial hip position at 90° flexion, a specific device was used Fig 2B [22, 23].

#### 2.6.2. Hamstring and low-back flexibility

The modified Back Saver Sit and reach test (BSSR) is a valid and reliable test widely used to evaluate the overall flexibility of the hamstring and lower back muscles in a unilateral way [24–26]. To perform the test, subjects were sat with the tested limb stretch on a bench. The untested leg was placed on the floor, with the knee at approximately 90°. Hereafter, a measuring tape was placed on the bench. Subjects aligned the sole foot of the tested leg with the 100-cm mark on the measuring tape. Then, subjects were asked to reach forward as far as possible while maintaining the knees, arms, and fingers extended. They must keep the two hands together with palms down, as shown in Fig 2A. The score was recorded as the most distant point reach on the measuring tape, subtracting it from the initial value (100 cm).

#### 2.6.3. Hamstring and gluteus maximus isometric strength

To evaluate the muscular isometric strength of the hamstring and gluteus maximus, a manual dynamometer (MicroFET2, Hoggan Scientific, Salt Lake City, Utah) was used. All measurements were performed three trials per muscle, in a prone position, on a padded bench.

For the hamstrings strength assessment, the subject foot was placed on a foam pad under the ankle, positioning the knee between 20–25 degrees of flexion [27]. Resistance was performed with a stable manual fixation on the back of the malleoli, placing there the dynamometer [28]. Then, knee flexion was requested with maximal progressive contraction for five seconds, without holding on to the stretcher Fig 2C. For the gluteus maximus strength assessment, a hip extension was requested, with 90 degrees of knee flexion. Resistance was exerted on the posterior and distal femur area, where the dynamometer was placed Fig 2D. The mean of the three trials was considered in the analysis. Data obtained were expressed in Newtons.

#### 2.6.4. Hamstring and gluteus maximus electrical activity

For electrical muscle activity a surface electromyograph (sEMG) (mDurance® Solutions SL, Granada, Spain) [21] was used. It is a validated system to measure muscle activity signals that cause the muscle to generate force and produce movement [29]. The muscles tested were gluteus maximus, biceps femoris and semitendinosus.

The mDurance® system consists of a Shimmer3 EMG unit (Realtime Technologies Ltd, Dublin, Ireland), which is a bipolar sEMG sensor for the acquisition of superficial muscle activity. Each Shimmer sensor is composed of two sEMG channels (sampling rate at 1,024 Hz, a bandwidth of 8.4 kHz, a signal resolution of 24 bits and overall amplification of 100–10,000 V/V). We used two sensors for each leg. The electrodes used were pre-gelled Ag/AgCl with a diameter of 10 mm, placed following the SENIAM recommendations. For the gluteus maximus record, electrodes were placed at 50% on the line between the sacral vertebrae and the greater trochanter. For biceps femoris at 50% on the line between the ischial tuberosity and the tibia’s lateral epicondyle and at 50% on the line between the ischial tuberosity and the tibia’s medial epicondyle for semitendinosus. The reference electrodes were placed on the fibular head and sacral bone.

Then with the mDurance (Android) mobile application, the data from the Shimmer unit is send to a online storage service, where the sEMG signals are stored and analyzed. EMGs data obtained for this study was the % root mean square of muscular activation.

Participants performed a maximum voluntary isometric contraction as a common method used to normalize EMGs signals [29]. This test was performed using the same procedure described above for hamstring and gluteus maximus strength and recorded to mDurance device. The electric activity data (sEMG) was simultaneously recorded on both limbs, while performing a Countermovement jump (CMJ) test explained bellow. Data obtained for this variable was the mean muscle activity during CMJ, as a percentage of maximum voluntary isometric contraction test.

#### 2.6.5. Lower limb performance

Countermovement jump is a vertical jump test widely used to evaluate lower limb muscle strength. It is characterized by an initial countermovement before the toe-off phase and provides information about the reactive strength and performance of the lower limbs Fig 2F [30]. For this propose, we used the My jump 2, a mobile application that employs the device’s camera to perform a frame-by-frame analysis in order to calculate different parameters related to the jump [31]. High reliability and accuracy of My Jump 2 compared to the gold standard (force plate) has been reported [32]. Following the methodology used by Balsalobre et al., the subject had to perform a maximum vertical jump without using the upper limbs. The applied force (Newtons) and jump height (cm) were measured with the My Jump 2 application [33]. The sEMG data recording and CMJ were synchronized to obtain muscle recruitment between the jump’s exact start and endpoint.

### 2.7. Intervention

The study was conducted at the Anatomical Laboratory of the Universitat Internacional de Catalunya between April and May 2021. A clinical researcher with more than fourteen years of experience with DF applied the protocol and was blind to the data collection process.

DF technique was applied with the necessary pressure to encompass the structure to be moved. Brief rapid traction was applied in a transverse direction with the hook fixed to the skin and underlying soft tissues.

**Fig 3:**
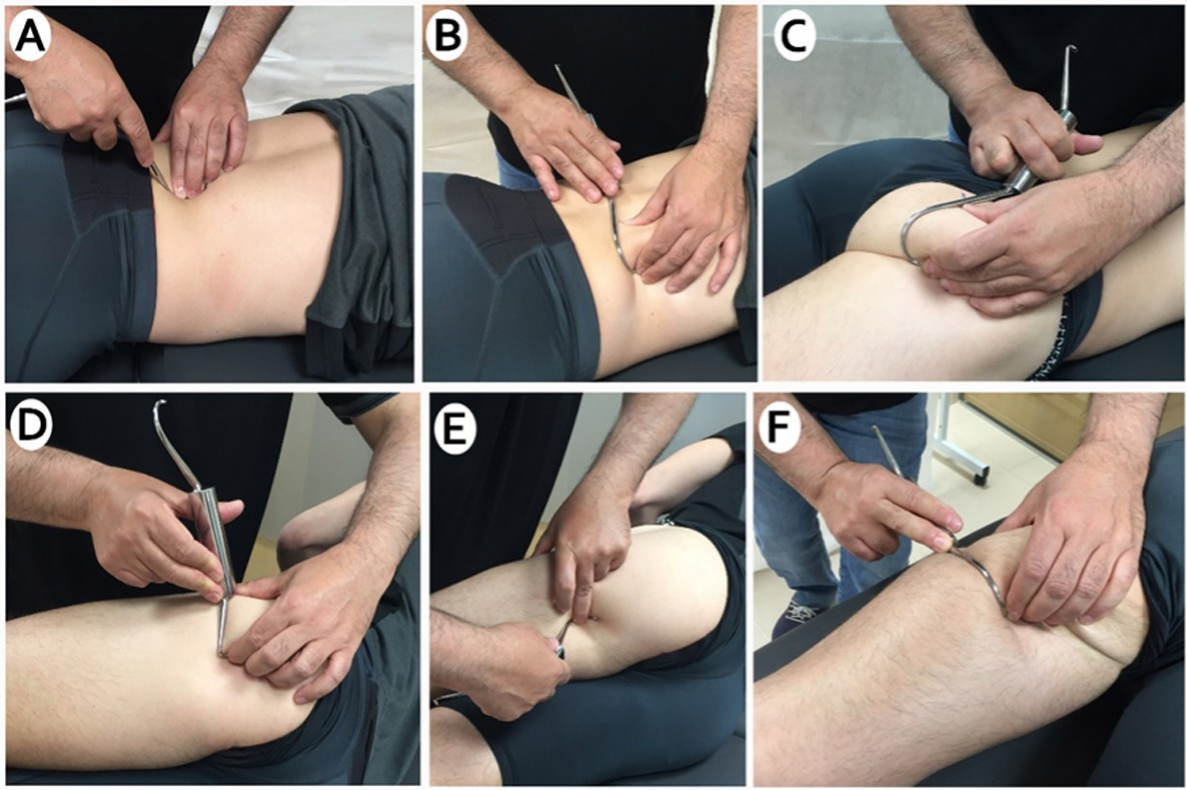
A-B) Diacutaneous fibrolysis to quadratus lumborum C) Diacutaneous fibrolysis to gluteal area, D) Diacutaneous fibrolysis between vastus externus and biceps fermoris E-F) Di-acutaneos fibrolysis hamstring area.

Acording to the cetriptal protocol, the following musculature and intermuscular septa received DF treatment on the experimental limb: quadratus lumbar, gluteus maximus, biceps femoris and semitendinosus. Participant was lying in the prone position. The application began with scraping and longitudinal strokes with the hook curvein the lumbar paravertebral region, quadratus lumborum and iliac crest.It was continued on the gluteal and trochanteric region with the classic technique. Then the protocol was followed by the posterior part of the tensor fascialis and vastus externus. And finally by the intermuscular septa between the vastus externus and biceps, biceps femoris and semitendinosus Fig 3. The time required for each diacutaneous session was about 10 minutes. The control limb was untreated. Room temperature was controlled between 22˚C-23˚C to avoid any alteration of the muscle properties [34].

### 2.8. Data analysis

For statistical analysis IBM SPSS Statistic 26.0 software was used to assess group differences in Hamstring length, flexibility, muscle strength, electrical activity and lower limb performance of success at each time interval. Descriptive analysis was carried out. Mean and standard deviation were calculated for quantitative variables. Frequencies were calculated for demographic and anthropologic qualitative variables. The Kolmogorov-Smirnov test was used to determine non-normal distribution of quantitative data (p > 0.05).

The linear mixed model was used to compare between-group and within-group changes over the three measurement periods with a auto-regressive covariance in a one-way mixed ANOVA. This model was performed for each dependent variable where the experimental/control limb was the between-subjects factor, and time was the within-subjects factor. If the assumption of sphericity was violated, the Greenhouse-Geisser correction was utilized for interpretation. When a statistically significant effect was noted, a post-hoc analysis was performed and the Bonferroni correction was used to adjust for multiple comparisons. All subjects originally enrolled were included in the final analysis as planned. Effect sizes (ES) were calculated using eta squared (ŋ^2^). Considering an effect size >0.14 as large; around 0.06 are medium; and <0.01 small. Losses and exclusions after randomization are explained in Fig 1. The statistical analysis was performed on an intention-to-treat basis (Little’s missing completely at random test and expectation maximization). The level of significance was set at p < 0.05.

## 3. Results

Seventy-three volunteers (Forty-six males and twenty-seven females) were recruited between April and May 2021 (involving 132 limbs). Seven female athletes did not meet the exclusion criteria (PKE>160°). The sample finally consisted of 66 athletes (66 experimental limbs and 66 control limbs). The mean age was 21.7 years (SD 3.5). There were no losses for the 30 minutes after treatment measurements (Fig 1). However, due to unknown technical issues in the recording of participants’ measurements, we had missing data for the muscle electrical activity outcomes in three subjects for the biceps femoris and semitendinosus muscles.

The sample’s demographic characteristics are summarized in Table 1. Football (14 athletes, 21.2%) followed by rugby with (8 athletes, 12.1%) were the most representative sports across the twenty registered disciplines. In relation to the dominance limb, the proportions were similar (50%). There were no adverse events with the treatments performed in the study. The comparative analysis of the study can be found in Tables 2–5).

**Table 1:** Characteristics of participants.

Sex:	
Men	46 (69.7%)
Women	20 (30.3%)
Age (years)	21.7 ± 3.5
Height (cm)	175.5 ± 8.34
Weight (kg)	70 ± 11.89
BMI (kg/m^2^)	22.71 ± 2.86

Descriptive results are shown as total number (percentage) and mean (±SD). Abbreviations: SD, Standard Deviation; n, number, %, percentage; cm, centimeters; kg, kilograms; m, meters.

**Table 2:** Intra-group analysis.

	T0	T1					T2				
Variables	Mean ± SD	Mean ± SD	Differences of means T0-T1	95% CI	p	ES(ŋ^2^)	Mean ± SD	Differences of means T0-T2	95% CI	p	ES(ŋ^2^)
Experimental Limbs											
Length (degrees)	143.26 ± 6.47	146.79 ± 9.24	3.53	[0.350; 6.711]	0.025	0.05	147.12 ± 8.46	3.86	[1.406; 6.321]	0.001	0.06
Flexibility (cm)	100.53 ± 9.66	103.13 ± 9.46	2.60	[1.596; 3.601]	0.001	0.02	103.36 ± 13.02	2.83	[0.043; 5.620]	0.045	0.02
Hamstring strength (N)	531.27 ± 146.61	549.15 ± 152.02	17.87	[-5.212; 40.957]	0.185	0.00	568.02 ± 161.90	36.74	[7.092; 66.392]	0.010	0.01
Gluteus max strength (N)	648.19 ± 187.81	695.73 ± 206.20	47.54	[7.552; 87.527]	0.014	0.01	734.81 ± 233.35	86.62	[43.023; 130.215]	0.001	0.04
Control Limbs											
Length (degrees)	142.88 ± 7.29	144.21 ± 9.30	1.33	[-1.821; 4.487]	0.908	0.01	143.41 ± 8.56	0.53	[-1.800; 2.860]	1.000	0.00
Flexibility (cm)	100.95 ± 10.30	102.87 ± 10.07	1.92	[0.847; 3.001]	0.001	0.01	104.58 ± 11.78	3.63	[1.835; 5.423]	0.001	0.03
Hamstring strength (N)	531.45 ± 130.17	535.35 ± 145.38	3.91	[-16.052; 23.862]	1.000	0.00	562.05 ± 160.26	30.61	[6.297; 54.915]	0.009	0.01
Gluteus max strength (N)	657.50 ± 196.60	663.88 ± 208.07	6.38	[-32-511; 45.279]	1.000	0.00	689.60 ± 212.53	32.10	[-3.194; 67.394]	0.087	0.01
Bilateral Test											
CMJ height (cm)	31.18 ± 9.13	30.60 ± 9.29	-0.58	[-2.125; 0.963]	1.000	0.00	31.59 ± 9.22	0.41	[-1.229; 2.053]	1.000	0.00
CMJ Force (N)	1650 ± 2403.69	1640.86 ± 2300.18	-9.19	[-64.526; 46.151]	1.000	0.00	1680.41 ± 2509.80	30.36	[-19.621; 80.340]	0.421	0.00

Abbreviations: SD, Standard Deviation; CI, Confidence interval; p, p-value; ES(ŋ^2^), Effect size eta square; cm, centimeters; N, Newtons; CMJ, Countermovement jump.

**Table 3:** Inter-group analysis.

	Difference T0-T1			Difference T0-T2		
Variable	Experimental Limbs	Control Limbs		Experimental Limbs	Control Limbs	
	Mean ± SD	Mean ± SD	p	Mean ± SD	Mean ± SD	p
Length (degrees)	3.53 ± 10.51	1.33 ± 10.43	0.077	3.86 ± 8.12	0.53 ± 7.70	0.001
Flexibility (cm)	2.60 ± 3.31	1.92 ± 3.56	0.162	2.83 ± 9.22	3.63 ± 5.93	0.544
Hamstring strength (N)	17.87 ± 76.31	3.90 ± 65.97	0.144	36.74 ± 98.02	30.61 ± 80.36	0.569
Gluteus max strength (N)	47.54 ± 132.19	6.38 ± 128.58	0.008	86.62 ± 144.12	32.10 ± 116.67	0.002

Abbreviations: SD, Standard Deviation; p, p-value; cm, centimeters; N, Newtons

**Table 4:** Intra-group electrical activity analysis.

T0			T1					T2				
		Mean ± SD	Mean ± SD	Differences of means T0-T1	95% CI	p	ES(ŋ^2^)	Mean ± SD	Differences of means T0-T2	95% CI	p	ES(ŋ^2^)
Experimental Limbs												
Gluteus max	MA %	16.85 ± 14.10	15.53 ± 12.54	-1.32	[-4.397; 1.755]	0.272	0.00	19.37 ± 24.16	2.52	[-2.300; 7.344]	0.610	0.00
Biceps femoris	MA %	14.79 ± 15.97	8.23 ± 10.70	-6.55	[-11.003; -2.100]	0.002	0.06	12.33 ± 25.24	-2.46	[-8.237; 3.323]	0.900	0.00
Semitendinosus	MA %	11.48 ± 14.12	7.96 ± 18.09	-3.51	[-10.014; 2.98]	0.565	0.01	11.22 ± 20.26	-0.25	[-7.080; 6.577]	1.000	0.00
Control Limbs												
Gluteus max	MA %	20.67 ± 37.32	15.94 ± 15.47	-4.73	[-12.178; 2.726]	0.372	0.01	15.96 ± 24.20	-4.71	[-12.006; 2.585]	0.352	0.01
Biceps femoris	MA %	14.74 ± 18.46	12.02 ± 13.12	-3.72	[-8.449; 1.011]	0.173	0.01	14.40 ± 28.31	-1.33	[-7.182; 4.513]	1.000	0.00
Semitendinosus	MA %	12.84 ± 14.50	9.54 ± 13.06	-3.30	[-8.973; 2.371]	0.472	0.01	10.90 ± 15.06	-1.94	[-5.413; 1.523]	0.519	0.00

Abbreviations: SD, Standard Deviation; CI, Confidence interval; p, p-value; ES (ŋ^2^), Effect size eta square; MA %, muscular activation percentage.

**Table 5:** Inter-group electrical activity analysis.

		Difference T0-T1			Difference T0-T2		
		Experimental Limbs	Control Limbs		Experimental Limbs	Control Limbs	
		Mean ± SD	Mean ± SD	p	Mean ± SD	Mean ± SD	p
Gluteus max	MA %	-1.32 ± 10.17	-4.73 ± 24.63	0.252	2.52 ± 15.94	-4.71 ± 24.11	0.100
Biceps femoris	MA %	-6.55 ± 14.71	-3.72 ± 15.64	0.170	-2.46 ± 19.10	-1.33 ± 19.32	0.550
Semitendinosus	MA %	-3.51 ± 21.48	-3.30 ± 18.75	0.954	-0.25 ± 22.57	-1.94 ± 11.46	0.571

Abbreviations: p, p-value; MA %, muscle activation percentage; SD, Standard Deviation.

### 3.1. Hamstring length

There were significant main effects in specific hamstring length for time: F = 3.399 (p = 0.036) and for group: F = 20.687 (p<0.001). There was also significant interaction between group and time: F = 5.111 (p = 0.007). These effects indicated that control group did not change over time whereas experimental group increased specific hamstring length over time (Table 2), and compared to the control group in the two moments of the study (Table 3).

For specific hamstring length, in the intergroup analysis there was a statistically significant difference favoring the experimental limbs with an improve of 3.86 degrees compared to the control limbs (p<0.001) only between T0 and T2 (Table 3).

In the intragroup analysis we only found statistically significant differences in the experimental limbs with an increase of 3.53 degrees at T1 (p = 0.025; ES: 0.05) and 3.86 degrees (p<0.001; ES: 0.06) at T2. In the control limbs, the increase in T1 was 1.33 degrees (p = 0.908; ES: 0.01) and 0.53 degrees in T2 (p = 1.000; ES: 0.00) (Table 2).

### 3.2. Hamstring and low-back flexibility

There were significant main effects in hamstring and low-back flexibility for time: F = 17.071 (p<0.001) but not for group: F = 0.528 (p = 0.470). There was no significant interaction between group and time: F = 0.977 (p = 0.379).

### 3.3. Hamstring and gluteus maximus strength

There were significant main effects in hamstring strength for time: F = 6.918 (p<0.001) but not for group: F = 1.142 (p = 0.289). There was no significant interaction between group and time: F = 0.985 (p = 0.376).

In gluteus maximus strength, there were significant main effects for time: F = 10.281 (p<0.001) and for group: F = 6.778 (p = 0.011). There was a significant interaction between group and time: F = 6.809 (p = 0.002). These effects indicated that control group did not change over time whereas experimental group increased gluteus maximus strength over time (Table 2), and compared to the control group in the two moments of the study (Table 3).

For gluteus maximus strength, in the intergroup analysis we found a statistically significant difference favouring the experimental limbs with a strength improve of 47.54 N (p = 0.008), compared to control limbs between T0 and T1, and an increase of 86.62 N (p = 0.002) between T0-T2 (Table 3). In the intragroup analysis, only the experimental limbs reached a statistically significant difference in both post treatment measurements. At T1 we found a strength mean increase of 47.57N (p = 0.014, ES: 0.01) and of 86.62 N at T2 (p<0.001; ES: 0.04) (Table 2).

### 3.4. Hamstring and gluteus maximus electrical activity

There were no significant main effects in hamstring electrical activity for time: Biceps femoris: F = 2.710 (p = 0.088); Semitendinosus: F = 2.149 (p = 0.121) but there were significant main effects for group, only for the Biceps femoris: Biceps femoris F = 5.006 (p = 0.029); Semitendinosus: F = 0.498 (p = 0.483). There were no significant interaction between group and time: Biceps femoris: F = 1.164 (p = 0.314); Semitendinosus: F = 0.209 (p = 0.812). In gluteus maximus electrical activity, there were significant main effects for time: F = 1.782 (p = 0.172) and for group: F = 0.035 (p = 0.852). There was significant interaction between group and time: F = 2.272 (p = 0.107).

### 3.5. Lower limbs performance

In the countermovement jump, we found a decrease of 0.58 cm in height jump and of 9.19 N in force jump at T1. However, at T2 the values of height and force jump slightly improved regarding to T0 (0.41 cm and 30.36 N, respectively) without statistical significance (Table 5).

## 4. Discussion

This study aims to evaluate the immediate and 30 minutes post-treatment effects of a single DF session, on hamstring length and flexibility as well as muscle strength, electrical activity and lower limb performance in athletes with hamstring shortening. Our results suggest that a single session of DF generates changes in hamstring length 30 minutes after the treatment, but does not affect lower back and hamstring flexibility, strength, muscle electrical activity and lower limb performance.

This is the first study evaluating the effects of DF on hamstring length, flexibility and active neuromuscular responses in athlets. Our results suggest a possible, non-significant, immediate increase in hamstring length and a significant increase 30 minutes after the treatment. Our results are in line with other clinical studies using DF. Barra et al.[6] found improvements in the ROM of flexion, abduction and internal rotation of the shoulder joint in patients with painful shoulder compared to a placebo group, after applying a single session of DF. However, they only evaluated its immediate effects. Further on, they conducted another randomized trial in patients with subacromial impingement syndrome with a three months follow up, where they found no significant differences between the groups [5]. The statistically improvements observed correspond to a length increase of 3.86 cm and, even this value is below the clinically relevant value established (4.00 cm), this difference is very small. This could be explained by the short intervention time. Thus it is possible that a longer intervention could achieve the clinically relevant changes. On the other hand, this improvements are related to 30 minutes after the intervention; it would be interesting to evaluate a longer-term follow-up in future research. It has been widely described how alterations on hamstring length can be a risk factor for hamstring strain injuries. It has been proposed that a greater muscle length may reduce the risk of muscle strain injuries, by allowing muscles to absorb more energy during lengthening [35, 36]. In that sense, other instrument assisted soft tissue mobilization (IASTM) techniques clinical trials have been performed on hamstring muscles. Markovic et al. [37] compared the acute effects of foam roller and fascial abrasion technique IASTM on hip and knee range of motion in football players, and they found a statistically significant increase in ROM compared to the control group. These improvements were kept 24 hours after the treatment. According to their findings, J. Gunn et al. [10] reported that IASTM is an effective technique specifically for treating tight and for improving ROM in hamstring muscles. Nevertheless, their intervention was combined with a stretching technique, unlike ours which was isolated. Therefore, our results suggest that the application of DF could be used as a therapeutic choice for preventive purposes to normalize the hamstring length in athletes with hamstring shortening. Changes on muscle length may be explained by the physiology behind other mobilization soft tissue techniques where mechanoreceptors are stimulated. Barnes et al. [38] pointed out that the tone decrease of striated muscle fibers is likely a central nervous system response to the tissue pressure. Although the changes observed in muscle length are in line with those obtained by other tissue mobilization techniques, more studies on DF are needed to accurately discuss our results, since the action mechanism of DF remains unclear.

Instead, we did not observe statistically significant differences between experimental and control limbs for global flexibility of the hamstrings and lower back. We believe that the fact of improving hamstring length but not the overall flexibility may be due to the use of the BSSR for the assessment. This test involves the structures of the lumbar region, and the treatment time was slightly less in the lower back than for the hamstrings. Nevertheless, there were statistically significant differences in the intra-group analysis immediately and 30 minutes after the application of DF, compared to the baseline data. We believe that these changes may also be due to the repetitive stretching effect implicit in the test itself.

Regarding the hamstring strength assessment, we found slight increases in the experimental limbs muscles, compared to control limbs, that were not statistically significant. Nevertheless gluteus maximus was the only one that reached statistically significant differences. This results could be in line with López de Celis et al. [4] study, where they observed a higher handgrip muscle strength when combined DF with physiotherapy treatment for patients with chronic lateral epicondylalgia. However, they applied six treatment sessions while in the present study only one 10-minute session was applied. Although it has not been studied in depth, we hypothesize that DF technique could produce strength improvements due to its mechanical effect. As suggested before, DF may allow to recover the normal slide of the different fascial layers, thus generating a better neuromuscular function, by eliminating the resistance between muscular septum [8]. Other manual therapy techniques have shown to improve strength. A possible explanation for this is that the shortened sarcomeres may be lengthened by the mechanical ischemic compressions involved in those techniques and that the reactive hyperemia may lead to improve oxygen supply and consequently better strength production [19]. Although our hypothesis has not been proved in our study we believe that a longer intervention, including more sessions or time per session, could provide more conclusive results.

On the other hand, as other studies of IASTM pointed before, we also highlight that DF technique has shown to improve the hamstring length with no concomitant detrimental effects on neuromuscular strength production [9]. This makes DF a technique applicable in sports contexts to address hamstring shortening without affecting performance capacity on the playing field or in training sessions.

We have not found conclusive results related to muscle electrical activity after applying the technique. Generally, although the differences were not statistically significant, we observed a decrease in the muscle activation percentage, after applying DF in the experimental limbs, but also in the control limbs, compared with the baseline data. We cannot rule out that the trend observed in the two groups of limbs can be explained by neurophysiological changes produced by manual therapy techniques, where effects can appear distally to the areas directly treated [39, 40]. However, our results differ from Leite et al. [8]. They evaluated the immediate effect of DF compared to placebo (sham-DF) on neuromuscular efficiency during an explosive force in plantar flexion, on lateral gastrocnemius, in athletes. They found a higher neuromuscular efficiency on the intervention group, which they attribute to myofascial tissue stiffness changes. They indicate that changes on viscoelasticity have an influence on force transmission, increasing resulting muscular strength [8].

Finally, no statistically significant changes were observed in the lower limb performance parameters during the CMJ. Nevertheless, we observed a small decrease in height and force jump immediately after DF treatment, and how these values were recovered, and even improved slightly, 30 minutes after the treatment when compared to baseline. Decreases in CMJ performance seems to be related to the application of flexibility improvements techniques, as reported before. Behm et al. [41] conducted a study in order to assess how different hamstring static stretching could affect to de jump performance. They found that independently of the static stretch applied resulted in significant impairments in jump height. Instead, although we have found hamstring length changes after DF, our results appear not to show an impact on the deterioration of jump performance. On the other hand, our results are in accordance with Zhang et al. [42]. They assessed the immediate effect of myofascial self-release of posterior muscle chain in recreationally active subjects and neither found effects on jump performance. As well as, Heon Lim et al. found that the vertical jump performance was increased in both groups, with no significant differences after a 5 minutes foam roller session on hamstring muscles [43]. Even so, we believe that we cannot draw conclusive results related to the lower limb performance. CMJ is a bilateral action and our intervention was performed on single limb only. Furthermore, as previously reported, while it is certain that the hamstrings are closely involved in CMJ, it requires instantaneous power of several muscular groups, thus it can be influenced by other muscle conditions besides the hamstrings.

According to the results of our study, it seems that the use of DF improve hamstring length. These findings are consistent with other studies that point to tissue viscoelasticity improvements using other manual or instrumental tissue mobilization techniques [44, 45]. Nevertheless, in contrast to them, it seems that FD has no relevant effect on active neuromuscular response parameters such as strength, muscle activity or countermovement jump.

## 5. Limitations

The present study has some limitations. We cannot assure that the recording order of the outcomes studied did no interfered with the results obtained. In addition, we have only evaluated the strength isometrically. Taking into account the possible release of adhesion between the different sliding planes, changes in the concentric or eccentric path could be obtained, which we suggest to be evaluated in future research. Furthermore, we did not apply a sham DF in the non-intervention group, as it was done in previous clinical trials, and the experimental group only received a single DF session. Also, the results refer to the immediate effects. Finally, the intra-subject design of our study ensures greater homogeneity of the groups, although we cannot rule out a central neurovegetative effect affecting both limbs. Thus, we recommend further research to address these points in the future.

## 6. Conclusion

A single session of diacutaneous fibrolysis in athletes with hamstring shortening, following the centripetal protocol for the posterior part of the thigh, produces improvements in hamstring length 30 minutes after, and in gluteus maximus strength immediately and 30 minutes after the treatment. It seems to have no effects on the overall hamstring and lower back flexibility or myoelectric activity. Importantly, the lower limb performance was not impaired after the treatment.

## Supporting information

S1 Checklist (PDF)

S2 Checklist (DOC)

S1 File (PDF)

S2 File (PDF)
