# Supplementary material for: Immediate effects of diacutaneous fibrolysis in athletes with hamstring shortening. A randomized within-participant clinical trial
Source: PLoS One. 2022 Jul 5;17(7):e0270218. doi: 10.1371/journal.pone.0270218 (PMC9255769; doi:10.1371/journal.pone.0270218)
Supplement: S2 Checklist — (DOC) [file pone.0270218.s002.doc]

**
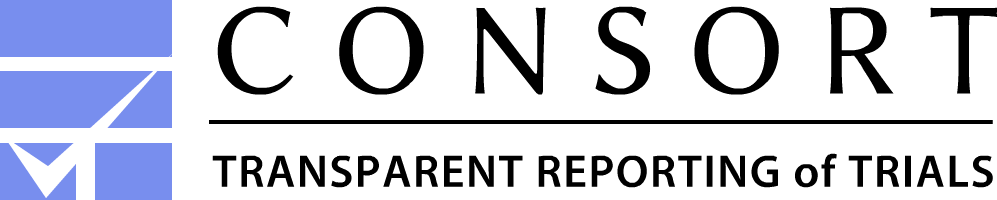
**

**VCONSORT 2010 Flow Diagram**

**T2 – 30 minutes Post-treatment**

**T1 – Post treatment**

**Enrollment**

**Analysis**

**Allocation**

**T0 – Pre treatment**

Assessed for eligibility (n=73)

Excluded (n= 7)

♦  Not meeting inclusion criteria (n=7)

PKE>160

Assessed to Experimental Limb (n= 66)

Assessed to Experimental Limb (n= 66)

Allocated to Experimental Limb (n= 66)

Assessed to Control Limb (n= 66)

Allocated to Control Limb (n= 66)

Assessed to Control Limb (n= 66)

Randomized (n= 66)

Analysed

BSSR (n=66)

PKE (n=66)

Dynamometry (n=66)

Contractile activity

Gluteus maximus (n= 63)

Biceps Femoris (n=63)

Semitendinosus (n=63)

Three data loss due to technical issues in the application

Countermovement jump (n=66)

Analysed

BSSR (n=66)

PKE (n=66)

Dynamometry (n=66)

Contractile activity

Gluteus maximus (n= 63)

Biceps Femoris (n=63)

Semitendinosus (n=63)

Three data loss due to technical issues in the application

Countermovement jump (n=66)
