# Supplementary material for: Immediate effects of diacutaneous fibrolysis in athletes with hamstring shortening. A randomized within-participant clinical trial
Source: PLoS One. 2022 Jul 5;17(7):e0270218. doi: 10.1371/journal.pone.0270218 (PMC9255769; doi:10.1371/journal.pone.0270218)
Supplement: S1 File — (PDF) [file pone.0270218.s003.pdf]

Doctoral Thesis Project Report

Effects of diacutaneous fibrolysis on neuromuscular response, flexibility, strength, and mechanosensitivity of the lower extremity posterior chain muscles in athletes.

Author: *Aida Cadellans Arróniz*
Director: *Dr. Carlos López de Celis*
Dr. Pere Ramón Rodríguez Rubio

Abstract

Introduction. Diacutaneous fibrolysis (DF) is a physiotherapeutic technique used to treat musculoskeletal disorders causing pain, restriction of movement and function, based on the Cyriax's deep transverse massage principles. Studies indicate that the main action mecanism could be mechanical, by releasing adhesions between the different tissue plans. It seems that the application of the technique can reorient the position of the extra and intrafusal fibers, improving circulation, muscle trophism, and muscle qualities such as contractibility or elasticity, properties involved in neuromuscular response.

Recent studies point to positive responses on different pathologies after the first session. However, the specific mechanisms of action have not been investigated in depth. No study has been found that evaluates its effects on the posterior chain where we believe that the neuromuscular response should be more evident to maintain the bipedal position.

Objective: To evaluate the immediate effects of the application of FD on the neuromuscular response (contractile muscle properties, muscle tone, mechanosensitivity, flexibility, strength and muscle activity) of the posterior chain of the lower extremity in athletes.

Methodology: The project will consist of two phases. The first one will study the passive neuromuscular response parameters and the second one the active neuromuscular response.

Randomized controlled clinical trial, with a masked evaluator. FD will be applied to posterior chain muscles at one lower extremity (experimental) while no technique will be applied to the other lower extremity (control). Including athletes who compete regularly. N=66 determined on the basis of the statistics of maximum radial displacement of the tensomiography of the biceps femoris muscle. The independent variables will be: age, sex, height, weight and sport practiced. The dependent variables will be; First phase: contractile muscle properties (tensomiography), muscle tone (myotonometry), mechanosensitivity (algometry). Second phase: flexibility (modified Back Sever Sit and Reach test and Passive Knee Extension test), muscle strength (dynamometry and "My Jump") and muscle activity (surface electromyography). Statistical analysis will be performed with SPSS v.26.0. Statistical, descriptive, intra-group and inter-extremity comparative analysis will be performed. The significance level will be $p<0.05$ with 95% confidence interval.

1. Background and state of art.

Diacutaneous fibrolysis (DF) is a physiotherapeutic technique used to treat locomotor system disorders that causing pain, restriction of movement or functional impairment. (1). It was developed by Kurt Ekman (2) attributing its action mechanism to be exclusively mechanical, breaking the connective tissue fibers that have formed adhesions, in order to regain the flatness between the different layers of tissue. Burnotte and Duby(1) also observed a circulatory and reflex effect. The technique is performed using metal hooks, ending in a spatula that allow a better distribution of pressure on the skin and greater depth precision, compared to the manual approach.

The application guide of the technique indicates it for the adhesions treatment as a consequence of trauma, post-surgical scar fibrosis or inflammatory lesions of the locomotor system (12).

The technique involves three successive phases; digital palpation, instrumental palpation and fibrolysis, where an additional traction is performed with the hook to free the tissue planes from possible adhesions.

Different studies hypostatizes that the main mechanism of action of FD could be mechanical allowing the release of the possible adhesions between the different tissular layers such as muscles, aponeurosis, tendons and others and has shown good clinical results (1-4). At the muscular level, it seems that the transversal application of the technique can reorient the position of the extra and intrafusal fibers. This myofascial tensions balance could improve the circulation, muscle trophism and muscle qualities such as contractibility and elasticity, factors involved in the neuromuscular response (5).

The neuromuscular response, also called neuromuscular function, is defined as the set of biomechanical and viscoelastic properties of the muscular and fascial tissue, which prepare the muscle for mechanical work, in response to the indications of the muscular and nervous system (5).

The parameters obtained through the evaluation of the neuromuscular response are useful to examine the tissue injury effects, as well as for monitor the treatment effects.

Recent studies point to positive responses with FD on different pathologies such as rangle of motion improvements in subacromial impingement syndrome (1), sensory conductivity improvements in symptomatic patients with carpal tunnel syndrome(3), pain decrease in patients with chronic lateral epycondylalgia (4) or athletes with anterior knee pain(2). They also indicate that positive responses appear from the first application of the technique(1). However, the specific action mechanism have not been investigated in depth. No studies have been found describing how the possible changes on the tissue affect mechanical and functional aspects such as range of motion and force.

Clinical studies show improvements in strength, pain intensity, range of motion, etc,(1-4) but it is not known whether this effect become as a consequence tissue tension changes or due to reflex aspects, as has been suggested before. There are no studies evaluating its effects on the posterior chain of athletes where we believe that the neuromuscular response must be more evident since, as indicated in the FD application manual (13), it is known that the hypersolicitation of the musculature favors biomechanical adhesions.

2. Hypotheses and objectives

Hypothesis: A single diacutaneous fibrolysis session to the lower extremity posterior chain musculature will produce improvements in neuromuscular response, flexibility and strength, and a decrease in mechanosensitivity in athletes.

Objectives:

- To evaluate the immediate and 30 minutes after effects of a single diacutaneous fibrolysis session on the neuromuscular response of the lower extremity posterior chain musculature in athletes through tensomiography, myotonometry, algometry, the modified Back Saver Sit and Reach test and Passive Knee Extension test, dynamometry, My jump application and surface electromyography.

3. To know which are the neuromuscular response parameters studied that show the most relevant changes after a single application of diacutaneous fibrolysis in order to compare them with other techniques in future studies.

3. Methodology.

PHASE 1 PASSIVE

Study design: Randomized controlled clinical trial, with masked assessor.

Randomization. Between lower limbs of each subject (random.org). The FD will be applied to the muscles of the posterior chain of the lower extremity: semitendinosus, biceps femoris, gluteus maximus quadratus lumborum and multifidus (lumbar) at one lower extremity (experimental limb) while the technique will not be applied to the other lower limb (control limb), regardless of its dominance.

Recruitment of the sample. Athletes who are part of the university community of the UIC, who compete officially or institutionally, who are federated or are listed in an official sports registry in a sport where lower limbs activity are predominance (athletics, cycling, soccer, rugby...).

As the main **inclusion** criteria, the participants must sign the informed consent form and must present a hamstring shortness considered as <160º in the PKE test (Passive Knee Extenxion) (14).

Subjects will be **excluded** if they had any type of contraindication related to the FD technique (poor skin or trophic condition, taking anticoagulants, suffering from an inflammatory process or having had a recent injury that did not allow regular sports practice).

Sample size.

N=66. Determined based on maximum radial displacement (Dm) of the tensiomiography of the biceps femoris muscle. Risc Alpha 0.05 and Beta risk lower than 0.2, assuming 15% of follow-up losses.

Study Outcomes.

Independent variables such as age, sex, height, weight and sport practiced will be collected.

Response variables: (Table 1).

- **Tensiomiography (TMG).** Tensomiography, measures in isometric conditions, the contractile muscle properties through an external electrical stimulus of controlled intensity. It presents a high level of reliability (r=0.93) and reproducibility. It allows information to be obtained on the state of structural fatigue, muscle activation, muscle tone or contractile properties of the muscle(5). Information is obtained on maximum radial displacement, contraction time, response time, contraction velocity, contraction maintenance time and relaxation time(6). We believe that

the clinical results observed in other studies such as increased strength or range of motion may be due to a decrease in muscle tone (1-4).

- **Myotonometry.** Myotonometry is a method of measuring muscle tone by examining the action and relaxation state of the muscle; by means of the Myoton, which evaluates the viscoelastic characteristics of the muscle. In resting State it release a mechanical impulse and, through an internal microprocessor, provides quantitative values of the viscoelastic properties of the tissues, such as muscle tone, stiffness and elasticity. It evaluates muscular tension, dynamic stiffness and decrease of the natural oscillation, relaxation time of the mechanical stress (7). The viscoelastic properties are modified by tissue mobilization techniques such as FD (12).

- **Mechanosensitivity.** It is defined as the ease with which nociceptive impulses can be activated as a consequence of the application of traction and/or compression forces to the tissue (14). Mechanical nociception is regulated by free nervous terminations stimulation (FNT) from mechanosensitive fibers with low response limits (A-delta and C fibers). Thus, depending on the intensity of the mechanical stimulus, the FNTs can behave as receptors of mechanical tension or as nociceptors in the presence excessive magnitude stimuli (15). Different clinical studies have demonstrated a decrease in pain after DF application, thus we are interested in knowing how mechanosensitivity can be modified (1-3).

Pressure algometry**.** Pressure algometry is a method that quantifies mechanosensitivity by applying a progressive compression mechanical stimuli to a located point on the body. For this purpose the trigger points are the ones used, in many studies. The algometers are easily accessible devices, and it's method has shown a high reliability (r= 0.80)(8).

TABLE 1. Outcomes response.

**Tensiomyography**	**Myotonometry**	**Algometry**
Maximum radial displacement (Dm)	Muscle torque (Hz)	Magnitude of applied force (kg)

Contraction time (Tc)	Dynamic stiffness (N/m)
Response time (Td)	Elasticity (Td)
Contraction speed (Vc)	Relaxation time
Contraction maintenance (Ts)	Creep (fluency)
Relaxation time (Tr)	

Procedure. The independent variables will be recorded at the beginning of the study, and the response variables at the beginning, immediately after the application of the technique, and 30 minutes after by a blinded evaluator following the protocols used in previous studies.

The pain on pressure (PPT) will be assess in the following posterior chain muscles: ischiosural, gluteus maximus, and quadratus lumborum, are measured through a pressure algometer.

A experienced physiotherapist will apply the FD technique in the lowe limb previously randomized.

PHASE 2 ACTIVE

The sample will be the same as in phase 1. The subjects will be convened 15 days after the first session. Outcomes register processes and intervention procedure will be the same described above.

Outcomes.

- **Flexibility**. Defined as the joint capacity to move fluidly through its entire range of motion (ROM). The presence of adhesions or a deficit in ROM is one of the main indications for the DF application and is therefore considered an important variable to be assess. Furthermore, clinical studies indicate an increase in joint range of motion after its application (1-4).

  The *modified Back Saver Sit and reach test* assesses the extensibility of the hamstring and lower leg muscles in a unilateral manner. It is a valid and reliable quantitative test (r=0.89-0.98), used in several studies (9).

  Through the *Passive Knee Extension* test, the flexibility of the hamstring musculature will be evaluated in a more specific way (15).

- **Neuromuscular strength and function.**

  The presence of adhesions is clinically evidenced through the biomechanical modification of contractile function (13) thus, we believe it is important to study the DF effects on strength and neuromuscular function.

Muscular strength. Strength is defined as the capacity to generate intramuscular tension to an external force. In the isometric muscular strength, there is muscular tension, but there is no movement or wringing of the muscle fibers. In the explosive strength, a contraction (concentric or eccentric) is performed at maximum velocity, displacing a small resistance.

The **isometric strength** will be evaluated by means of a digital dynamometer (Microfeet 2) and the **explosive strength** will be evaluated through the jump, involving the concentric muscular strength, during the propulsion, and eccentrically, during the reception, through the "My jump". Mobile application that shows high reproducibility and reliability in vertical jumps, in comparison with force platforms. (10).

Due to displacement between the different tissue plans improvements that FD seems to produce, we consider interesting to evaluate how strength can be modified in isometric but also explosive modalities, since both present differences in the displacement of muscle fibers during contraction. Furthermore, previous studies have already demonstrated strength improvements in pain-free grip strength in patients with chronic lateral epicodilalgia after FD (4).

- **Neuromuscular function (NMF).** Muscle contraccion must be activated by the motor nerve fibers, which come from the central nervous system, to the different muscle innervated zones.

Surface electromyogram (SEMG). Is a noninvasive device used for the muscle activity analysis, which allows information from the muscles during rest or movement.

It has been used in pathological processes but also as a support in clinical interventions. It allows to determine if a muscle is involved in a certain action, and also to detect how the muscles coordinate with each other (11). We are interested to know the muscle activity during a functional movement,

by means of SEMG. Functional improvements have been reported in clinical studies, both in the upper and lower extremities, after the application of DF (2)(4).

Phase 2 response variables:(table 2).

TABLE 2. Response Outcomes.

**BACK SAVER SIT AND REACH**	**DINAMOMETRY** (microfet 2)	**MY JUMP**	**SEMG** (Mdurance pro-motion)
Hamstring Flexibility (cm)	Hamstring and gluteus isometric muscular strength (Kg)	Explosive muscular strength (vertical height and power peak)	semitendinosus, biceps femoris and multifidus (lumbar area)<br><br>Maximum voluntary contraction (MVC)<br><br>Root mean square value (RMS)<br><br>Mean frequency (MEF)<br><br>Conduction velocity (CV)<br><br>Innervated zones (IZ)

Procedure. A blind evaluator will take recorded the outcomes.

Statistical analysis. It will be performed with the SPSS Statistics v.26.0 program. Descriptive statistics will be calculated. Qualitative variables will be expressed as number and percentage, and quantitative variables and their differences as mean and standard deviation.

The normal distribution of quantitative variables will be checked by means of the Kolmogorov-Smirnov test (Lilliefors corrections) or the Shapiro-Wilk test, depending on the sample size. The homogeneity of the data between extremes will be checked at the beginning. For qualitative variables, the Chi-square or Fisher's exact statistic will be used otherwise. For quantitative variables, the Student t test or the Wilcoxon test will be used according to the results of the normality test.

Homoscedasticity will be checked with Levene's test or sphericity according to Mauchly's test.

An intra-group analysis will be performed using the repeated samples ANOVA test with the Bonferroni post hoc test and, if normality is not met, the Friedman test with the Wilcoxon pairwise comparison. In the comparison between groups, a comparison of the differences between the different periods will be made and analyzed using the Student t test or the Wilcoxon test. In the case

of qualitative variables in the comparison between groups, the Chi-square test or Fisher's exact statistic will be used. $p<0.05$ with a 95% confidence interval.

Ethical aspects.

- Prior approval of the project by the Research Ethics Committee (REC) UIC-Barcelona.
- The guidelines of the Helsinki Declaration (1973) will be respected.
- The signature of the informed consent will be requested and the Organic Law 3/2018, of December 5, on the Protection of Personal Data will be respected.

1. Pla de treball (cronograma)

	2019											
PAHSES	JANUARY	FEBRUARY	MARCH	APRIL	MAY	JUNE	JULY	AUGUST	SEPTEMBER	OCTOVER	NOVEMBER	DECEMBER
I. Research Plan												
Research and literature review									■	■	■	■
Problem statement									■	■	■	■
Project writing									■	■	■	■
Submission to the director												
II. Doctorate Academic Committee												
CAD project submission												
CED presentation												
CED Modifications												
III. Theoretical framework												
Drafting Theoretical framework/Methodology												
IV. Research development												
CER project submission												
Training procedure												
Recruitment of the sample												
Data collection												
Data analysis												
Writing results												
Corrections												
V. Conclusions												
Writing conclusions												
Corrections												
VI. Doctoral thesis reading procedures												
Send thesis draft to the director												
Corrections												
Thesis submission												
Administrative procedures for thesis defense												
Thesis defense												

	2020											
PAHSES	JANUARY	FEBRUARY	MARCH	APRIL	MAY	JUNE	JULY	AUGUST	SEPTEMBER	OCTOVER	NOVEMBER	DECEMBER
I. Research Plan												
Research and literature review												
Problem statement												
Project writing	■	■										
Submission to the director	■	■										
II. Doctorate Academic Committee												
CAD project submission		■	■									
CED presentation			■									
CED Modifications				■								
III. Theoretical framework												
Drafting Theoretical framework/Methodology				■	■	■	■	■	■	■	■	■
IV. Research development												
CER project submission				■	■							
Training procedure				■	■	■	■					
Recruitment of the sample									■	■	■	■
Data collection									■	■	■	■
Data analysis												
Writing results												
Corrections												
V. Conclusions												
Writing conclusions												
Corrections												
VI. Doctoral thesis reading procedures												
Send thesis draft to the director												
Corrections												
Thesis submission												
Administrative procedures for thesis defense												
Thesis defense												

	2021											
PAHSES	JANUARY	FEBRUARY	MARCH	APRIL	MAY	JUNE	JULY	AUGUST	SEPTEMBER	OCTOVER	NOVEMBER	DECEMBER
I. Research Plan												
Research and literature review												
Problem statement												
Project writing												
Submission to the director												
II. Doctorate Academic Committee												
CAD project submission												
CED presentation												
CED Modifications												
III. Theoretical framework												
Drafting Theoretical framework/Methodology	■	■	■	■	■	■	■		■	■	■	■
IV. Research development												
CER project submission												
Training procedure												
Recruitment of the sample												
Data collection	■	■	■	■	■	■	■		■	■	■	■
Data analysis												
Writing results												
Corrections												
V. Conclusions												
Writing conclusions												
Corrections												
VI. Doctoral thesis reading procedures												
Send thesis draft to the director												
Corrections												
Thesis submission												
Administrative procedures for thesis defense												
Thesis defense												

	2022											
PAHSES	JANUARY	FEBRUARY	MARCH	APRIL	MAY	JUNE	JULY	AUGUST	SEPTEMBER	OCTOVER	NOVEMBER	DECEMBER
I. Research Plan												
Research and literature review												
Problem statement												
Project writing												
Submission to the director												
II. Doctorate Academic Committee												
CAD project submission												
CED presentation												
CED Modifications												
III. Theoretical framework												
Drafting Theoretical framework/Methodology												
IV. Research development												
CER project submission												
Training procedure												
Recruitment of the sample												
Data collection												
Data analysis	■	■	■									
Writing results				■	■	■						
Corrections					■	■						
V. Conclusions												
Writing conclusions						■	■					
Corrections						■	■					
VI. Doctoral thesis reading procedures												
Send thesis draft to the director							■		■			
Corrections										■		
Thesis submission										■	■	
Administrative procedures for thesis defense										■	■	
Thesis defense												■

4. Limitations

- The neuromuscular response will be performed on subjects without pathology.
- The specificity and specificity of the characteristics of the sample does not represent the totality of the target population susceptible to FD treatment.
- Short-term evaluation, after a single session.

5. Experience of thesis directors in the area of knowledge.

Dr. Carlos López de Celis: PhD from the University of Zaragoza (2016), Master in Primary Care Research, UMH and UAB (2009-2012). Professor Lector of the UIC Barcelona, and titular Physiotherapist of the ICS. Principal investigator of the Study Group in Locomotor Pathology in Primary Care (GEPALAP) accredited by IDIAP Jordi Gol.

- Effectiveness of Diacutaneous Fibrolysis for the treatment of chronic Lateral Epicondylalgia. A randomized clinical trial. López de Celis C, Barra López ME, González Rueda V, Bueno Gracia E, Rodríguez Rubio PR, Tricás Moreno JM. Clin Rehabil. 2018;32(5):644-653. (Q1)
- Effectiveness of Diacutaneous Fibrolysis for the treatment of subacromial impingement syndrome: A randomised controlled trial. Barra López ME, López de Celis C, Fernández Jentsch G, Raya de Cárdenas L, Lucha López MO, Tricás Moreno JM. Manual Therapy. 2013;18(5);418-424. (Q2)
- The immediate effects of diacutaneous fibrolysis on pain and mobility in patients suffering from painful shoulder: a randomized placebo-controlled pilot study. Barra López ME, López de Celis C, Fernández Jentsch G, Murilo Barrios E, Villar Mateo E, Raya Cardenas L. Clinical Rehabilitacion. 2011;25:339-348. (Q1)

Dr. Pere Ramón Rodriguez Rubio: Doctor in Physiotherapy UIC-Barcelona (2014). Màster Universitari en Fisioteràpia i Evidència Científica. (2012). Assistant Professor and sub-director of the Department of Physiotherapy at UIC-Barcelona.

- Effectiveness of Diacutaneous Fibrolysis for the treatment of chronic Lateral Epicondylalgia. A randomized clinical trial. López de Celis C, Barra López ME, González Rueda V, Bueno Gracia E, Rodríguez Rubio PR, Tricás Moreno JM. Clin Rehabil. 2018;32(5):644-653. (Q1)

5. Financing and conflict of interest.

It is foreseen to apply for competitive grants during the process.

6. Main bibliography

1.- Barra ME, López C, Fernández G, Murillo E, Villar E, Raya L. The immediate effects of diacutaneous fibrolysis on pain and mobility in patients suffering from painful shoulder: A randomized placebo-controlled pilot study. Clin Rehabil. 2011;25(4):339–48.

2.- Fanlo-Mazas P, Bueno-Gracia E, de Escudero-Zapico AR, Tricás-Moreno JM, Lucha-López MO. The Effect of Diacutaneous Fibrolysis on Patellar Position Measured Using Ultrasound Scanning in Patients With Patellofemoral Pain Syndrome. J Sport Rehabil. 2018;28(6):564–9.

3.- Jiménez Del Barrio S, Estébanez de Miguel E, Bueno Gracia E, Haddad Garay M, Tricás Moreno JM, Hidalgo García C. Effects of diacutaneous fibrolysis in patients with mild to moderate symptomatic carpal tunnel syndrome: a randomized controlled trial. Clin Rehabil. 2018;32(12):1645-1655.

4.- López-de-Celis C, Barra-López ME, González-Rueda V, Bueno-Gracia E, Rodríguez-Rubio PR, Tricás-Moreno JM. Effectiveness of diacutaneous fibrolysis for the treatment of chronic lateral epicondylalgia: a randomized clinical trial. Clin Rehabil. 2018;32(5):644–53.

5.- Martín-Rodríguez S, Loturco I, Hunter AM, Rodríguez-Ruiz D, Munguia-Izquierdo D. Reliability and Measurement Error of Tensiomyography to Assess Mechanical Muscle Function: A Systematic Review. J Strength Cond Res. 2017;31(12):3524-3536.

6.- Lohr C, Schmidt T, Medina-Porqueres I, Braumann KM, Reer R, Porthun J. Diagnostic accuracy, validity, and reliability of Tensiomyography to assess muscle function and exercise-induced fatigue in healthy participants. A systematic review with meta-analysis. J Electromyogr Kinesiol. 2019;47:65-87.

7.- Dellalana LE, Chen F, Vain A, Gandelman JS, Põldemaa M, Chen H, et al. Reproducibility of the durometer and myoton devices for skin stiffness measurement in healthy subjects. Ski Res Technol. 2019;25(3):289–93.

8.- Hven L, Frost P, Bonde JP. Evaluation of Pressure Pain Threshold as a Measure of Perceived Stress and High Job Strain. PLoS One. 2017;12(1):e0167257.

9.- Hui SSC, Yuen PY. Validity of the modified back-saver sit-and-reach test: A comparison with other protocols. Med Sci Sports Exerc. 2000;32(9):1655–9.

10.- Cruvinel-Cabral RM, Oliveira-Silva I, Medeiros AR, Claudino JG, Jiménez-Reyes P, Boullosa DA. The validity and reliability of the "My Jump App" for measuring jump height of the elderly. PeerJ. 2018;6:e5804.

11.- Bussey MD, Aldabe D, Adhia D, Mani R. Reliability of surface electromyography activity of gluteal and hamstring muscles during sub-maximal and maximal voluntary isometric contractions. Musculoskelet Sci Pract. 2018;34:103-107.

12.- Tricás JM, Lucha O, Duby P. Fibrolisis Diacutánea según el concepto de Kurt Ekman. Zaragoza: Asociación Española de Fibrolisis Diacutánea; 2010.

13. da Silva Dias R, Gómez-Conesa A. Síndrome de los isquiotibiales acortados. Fisioterapia. 2008;30(4):186–93.

14.- Butler D. The sensitive nervous system. Noigroup Publications. 2006

15.- Leandro Hernán Caamaño Barrios. Directores. Dr. Ricardo Ortega Santiago Dr. Fernando Galán del Río. Evaluación de las alteraciones de la mecanosensibilidad, puntos gatillo miofasciales y movilidad neural en mujeres con cefalea tensional frecuente episódica. Unversidad Rey Juan Carlos. Madrid. 2020.

16.- Chen J, Choi MSE, Kim MSM. Immediate Effect of Intermittent Versus Continuous Hamstring Static Stretching on the Muscle Tone and Range of Motion. J Korean Soc Phys Med 2019;14(4):19-27
