# Supplementary material for: Immediate effects of diacutaneous fibrolysis in athletes with hamstring shortening. A randomized within-participant clinical trial
Source: PLoS One. 2022 Jul 5;17(7):e0270218. doi: 10.1371/journal.pone.0270218 (PMC9255769; doi:10.1371/journal.pone.0270218)
Supplement: S2 File — (PDF) [file pone.0270218.s004.pdf]

Memòria del Projecte de Tesi Doctoral

Efectes de la fibròlisi diacutània en la resposta neuromuscular, la flexibilitat, la força, i la mecanosensibilitat sobre els músculs de la cadena posterior de l'extremitat inferior en esportistes.

Autora: *Aida Cadellans Arróniz*
Director: *Dr. Carlos López de Celis*
Dr. Pere Ramón Rodríguez Rubio

Resum

Introducció. La fibròlisi diacutània (FD) és una tècnica fisioterapèutica que s'utilitza per tractar afectacions de l'aparell locomotor, que cursen amb dolor, restricció del moviment i funcionalitat, basada en els principis del massatge transvers profund de Cyriax. Estudis indiquen que el principal mecanisme d'acció, podria ser mecànic, alliberant possibles adherències entre els diferents plans de lliscament tissulars. L'aplicació de la tècnica pot reorientar la posició de les fibres extra i intrafusals afavorint la circulació, millorant el trofisme muscular, i les qualitats musculars com ara la contractibilitat o l'elasticitat, factors implicats en la resposta neuromuscular.

Estudis recents apunten respostes positives sobre diferents patologies després de la primera sessió. No obstant, els mecanismes d'acció específics, no han estat investigats en profunditat. No s'ha trobat cap estudi que avaluï els seus efectes sobre la cadena posterior on creiem que la resposta neuromuscular ha de ser més evident per mantenir la posició bípeda.

Objectiu: Avaluar els efectes immediats de l'aplicació de la FD sobre la resposta neuromuscular (propietats musculars contràctils, to muscular, la mecanosensibilitat flexibilitat, força i activitat muscular) de la cadena posterior de l'extremitat inferior, en esportistes.

Metodologia: El projecte constarà de dues fases. La primera s'estudiaran els paràmetres de resposta neuromuscular passiva i la segona la resposta neuromuscular activa.

Assaig clínic aleatoritzat controlat, amb avaluador emmascarat. S'aplicarà la FD a musculatura de la cadena posterior a una extremitat inferior (experimental) mentre que, a l'altra extremitat inferior, no se li aplicarà cap tècnica (control). S'inclouran esportistes que competeixin regularment. N=66 determinada en base als estadístics desplaçament radial màxim de la tensomiografia del múscul bíceps femoral. Les variables independents seran: edat, sexe, talla, pes i esport practicat. Les variables dependents seran; Primera fase: propietats musculars contràctils (tensomiografia), to muscular (miotonometria), mecanosensibilitat (algometria). Segona fase: flexibilitat (test modificat Back Sever Sit and Reach i Passive Knee Extension test), força muscular (dinamometria i "My Jump") i activitat muscular (electromiografia de superfície). L'anàlisi estadístic es realitzarà amb SPSS v.26.0. Es realitzarà un anàlisis estadístic, descriptiu, comparatiu intra-grupal i comparatiu entre extremitats. El nivell de significació serà $p<0.05$ amb 95% d'interval de confiança.

1. Antecedents i estat actual del tema.

La fibròlisi diacutània (FD) és una tècnica fisioterapèutica que s'utilitza per tractar afectacions de l'aparell locomotor, que cursen amb dolor, restricció del moviment o funcionalitat(1). Va ser desenvolupada per Kurt Ekman(2) atribuint el seu mecanisme d'acció exclusivament mecànic, trencant les fibres de teixit conjuntiu que hagin format adherències, per recuperar el lliscament entre les diferents capes de teixit. Burnotte i Duby(1) també observaven un efecte circulatori i reflex. La tècnica es realitza mitjançant uns ganxos metàl·lics, que acaben en una espàtula i permeten una millor distribució de la pressió sobre la pell, major precisió i profunditat, en comparació amb l'abordatge manual.

La guia d'aplicació de la tècnica la indica per al tractament d'adherències com a conseqüència d'un traumatisme, una fibrosi cicatricial post-quirúrgica o àlgies inflamatòries de l'aparell locomotor (12).

La tècnica comporta tres fases successives; palpació digital, palpació instrumental i fibròlisi, on es realitza una tracció suplementària amb el ganxo per alliberar els plans tissulars de les possibles adherències.

Diferents estudis senyalen la hipòtesi que el principal mecanisme d'acció de la FD podria ser mecànic, i que permetria alliberar les possibles adherències entre els diferents plans de lliscament tissulars com ara els músculs, l'aponeurosi, tendons i altres amb bons resultats clínics (1-4). A nivell muscular, es coneix que l'aplicació transversal de la tècnica pot reorientar la posició de les fibres extra i intrafusals. Aquest equilibri de les tensions miofascials afavoreix la circulació millorant el trofisme muscular, i les qualitats musculars com la contractibilitat o l'elasticitat, factors implicats en la resposta neuromuscular (5).

La resposta neuromuscular, també anomenada funció neuromuscular, es defineix com el conjunt de propietats biomecàniques i viscoelàstiques del teixit muscular i fascial, que preparen al múscul per un treball mecànic, en resposta a les indicacions del sistema muscular i nerviós (5).

Els paràmetres obtinguts a través de l'avaluació de la resposta neuromuscular, son útils per examinar els efectes d'una lesió en el teixit, així com la seva millora després de l'aplicació d'un tractament.

Estudis recents apunten respostes positives amb la FD sobre diferents patologies com ara la millora de mobilitat articular en el síndrome de impingement subacromial (1), millora de la conductivitat sensorial en pacients simptomàtics amb el síndrome del túnel carpià(3), disminució del dolor en pacients amb epicondiàlgia crònica(4) o esportistes que cursen amb dolor anterior del genoll(2). També indiquen que les respostes positives es produeixen des de la primera aplicació de la tècnica(1). No obstant, els mecanismes d'acció específics, no s'han investigat en profunditat. No s'han trobat

estudis que descriguin com els possibles canvis sobre el teixit, repercuteixen sobre aspectes mecànics i funcionals com ara, rang de moviment i la força.

Estudis clínics evidencien millores a la força, intensitat del dolor, rang de moviment, etc,(1-4) però no es coneix si aquest efecte és com a conseqüència del canvi de tensió del teixit o per aspectes reflexos, com s'ha suggerit en alguns estudis. No hi ha cap estudi que avaluï els seus efectes a la cadena posterior d'esportistes on creiem que la resposta neuromuscular ha de ser mes evident ja que, tal i com indica el manual d'aplicació de la FD (13), és conegut que la hipersol.licitació de la musculatura propicia adherències del tipus biomecàniques.

2. Hipòtesi i objectius

Hipòtesi: S'espera que una sola aplicació de fibròlisi diacutània a la musculatura de la cadena posterior de l'extremitat inferior, produeixi millores sobre la resposta neuromuscular, la flexibilitat i la força, i una disminució de la mecanosensibilitat en esportistes.

Objectius:

- Avaluar els efectes immediats i al cap de 30 minuts de l'aplicació de la fibròlisi diacutània sobre la resposta neuromuscular de la musculatura de la cadena posterior de l'extremitat inferior en esportistes a través de la tensomiografia, miotonometria, algometria, el test modificat Back Saver Sit and Reach i Passive Knee Extension test, la dinamometría, l'aplicació My jump i l'electromiografia de superfície.

- Conèixer quins son els paràmetres de resposta neuromusculars estudiats que presenten els canvis més rellevants després d'una sola aplicació de la fibròlisi diacutània per tal de, en futurs estudis, poder-los comparar amb altres tècniques.

3. **Metodologia.**

FASE 1 PASIVA

Disseny de l'estudi: Assaig clínic aleatoritzat controlat, amb avaluador emmascarat.

Aleatorització. Entre extremitats inferiors de cada subjecte (random.org). S'aplicarà la FD als músculs de la cadena posterior de la extremitat inferior: semitendinós, bíceps femoral, gluti major quadrat lumbar i multífids (lumbar) a una extremitat inferior (extremitat experimental) mentre que, a l'altra extremitat inferior, no se li aplica la tècnica (extremitat control), independentment de la seva dominància.

Reclutament de la mostra. Esportistes que formin part de la comunitat universitària de la UIC, que competeixin de forma oficial o insitituacional, que estiguin federats o constin en algun registre esportiu oficial en algun esport on l'activitat predominant se centri en el tren inferior (atletisme, ciclisme, futbol, rugbi...).

Com a **criteri d'inclusió** principal els participats hauran de firmar el consentiment informat i hauran de presentar un escurçament de la musculatura isquiosural considerant-se com a tal <160º en el test PKE (Passive Knee Extenxion) (14).

Quedaran **exclosos** de la mostra aquells subjectes que presentin algun tipus de contraindicació relacionada amb la tècnica de FD (mal estat cutani o tròfic, que prenguin anticoagulants, que pateixin algun procés inflamatori o que hagin tingut alguna lesió recent que no permeti la practica esportiva habitual).

Mida de la mostra.

N=66. Determinada en base als estadístics de desplaçament radial màxim (Dm) de la tensiomiografia pel múscul bíceps femoral. Risc Alfa 0.05 i risc Beta inferior a 0.2, assumint un 15% de pèrdues de seguiment.

Variables d'estudi. Es recolliran les variables independents com edat, sexe, talla, pes i esport practicat.

Variables resposta:(Taula 1).

- **Tensomiografia (TMG).** La tensomiografia, mesura en condicions isomètriques, les propietats musculars contràctils a través d'un estímul elèctric extern, d'intensitat controlada. Presenta un alt nivell de fiabilitat (r=0,93) i reproductivitat. Permet obtenir informació sobre l'estat de fatiga

estructural, activació muscular, to muscular o propietats contràctils del múscul(5). S'obté informació sobre el desplaçament radial màxim, temps de contracció , temps de resposta, velocitat de contracció, temps de manteniment de la contracció i temps de relaxació(6). Creiem que els resultats clínics observats en altres estudis com l'augment de la força o el rang de mobilitat es poden deure a una disminució del to muscular (1-4).

- **Miotonometria.** La miotonometria, és un mètode de mesura del to muscular on s'examina l'estat d'acció i relaxació del múscul; mitjançant el *Myoton*, que valora les característiques viscoelàstiques del múscul, en estat de repòs, alliberant un impuls mecànic i, a través d'un microprocessador intern, ofereix valors quantitatius de les propietats viscoelàstiques dels teixits, com ara el to muscular, la rigidesa i la seva elasticitat. Avalua, to muscular, rigidesa dinàmica i disminució de la oscil·lació natural, temps de relaxació del estres mecànic (7). Les propietats viscoelàstiques es veuen modificades per les tècniques de mobilització del teixit com ara es la FD (12).

- **Mecanosensibilitat.** Es defineix com la facilitat amb la que es poden activar els impulsos nociceptius com a conseqüència de l'aplicació de forces de tracció i/o compressió al teixit (14). La nocicepció d'origen mecànic esta regulada per l'estimulació de les terminacions nervioses lliures (TNL) procedents de fibres de naturalesa mecanosensible amb llindars de resposta baixos (fibres A-delta i C). Així, en funció de la intensitat de l'estímul mecànic les TNL es poden comportar com a receptors de tensió mecànica o com a nociceptors davant d'estímuls de magnitud excessiva (15). Diferents estudis clínics han demostrat una disminució del dolor després de l'aplicació de la FD, és per això que ens interessa conèixer com es pot veure modificada la mecanosensibilitat (1-3).

Algometria de pressió. L'algometria de pressió, és un mètode que quantifica la mecanosensibilitat, aplicant un estímul mecànic de compressió progressiva sobre un punt localitzat en el cos. En nombrosos estudis, els punts gallets, son els utilitzat per aquest fi. Els algòmetres son dispositius de fàcil accessibilitat, i es tracta d'un mètode que ha presentat una alta fiabilitat (r= 0,80)(8).

TAULA 1. Variables de resposta estudiades.

TENSOMIOGRAFIA	MIOTONOMETRIA	ALGOMETRIA
Desplaçament radial màxim (Dm)	To muscular (Hz)	Magnitud de força aplicada (kg)
Temps de contracció (Tc)	Rigidesa dinàmica (N/m)	

Temps de resposta (Td)	Elasticitat
Velocitat de contracció (Vc)	Temps de relaxació
Manteniment de contracció (Ts)	Creep (fluència)
Temps de relaxació (Tr)	

Procediment. Les variables independents es registraran al inici de l'estudi, i les variables de resposta, al inici, immediatament després de l'aplicació de la tècnica, i 30 minuts després de la aplicació.

Un avaluador emmascarat prendrà els valors de les variables a estudiar.

Per la tensomiografia, se seguiran els protocols utilitzats en publicacions anteriors.

El llindar del dolor a la pressió (PPT) dels músculs de la cadena posterior: isquiosurals, gluti major, i quadrat lumbar, es mesuraran a través d'un algòmetre de pressió.

Un fisioterapeuta experimentat en la tècnica de FD, serà l'encarregat d'aplicar-la a l'extremitat que ha estat prèviament aleatoritzada.

FASE 2 ACTIVA

La mostra serà la mateixa que a la fase 1 citant als subjectes a partir de 15 dies després de la primera sessió.

Variables.

- **Flexibilitat.** És la capacitat d'una articulació per moure's fluidament per tota la seva amplitud de moviment (ROM). La presència d'adherències o un dèficit en el ROM és una de les indicacions principals per l'aplicació de la tècnica i per això es considera una variable imprescindible. A més, estudis clínics indiquen un augment del rang de moviment articular després de la seva aplicació (1-4).

  Back Saver Sit and reach test modificat, avalua l'extensibilitat de la musculatura isquiosural i part baixa de l'esquena de manera unilateral. És una eina quantitativa, vàlida i fiable (r=0,89-0,98), utilitzada en nombrosos estudis(9).

  A través del *Passive Knee Extension test* s'avaluarà d'una forma més específica la flexibilitat de la musculatura isquiosural (15).

- **La força i la funció neuromuscular.**

  La presencia d'adherències s'evidencia clínicament a través de la modificació biomecànica de la funció contràctil (13) i es per això que creiem rellevant poder estudiar els efectes de la FD sobre la força i la funció neuromuscular.

  Força muscular. La força es defineix com la capacitat de generar tensió intramuscular, per vèncer o oposar-se a una força externa.

  La **força muscular isomètrica**, existeix tensió muscular, però no es produeix un moviment ni escurçament de les fibres musculars, per no vèncer cap resistència.

  La **força explosiva,** es realitza una contracció (concèntrica o excèntrica) a la màxima velocitat, desplaçant una resistència petita.

  La força isomètrica, s'avaluarà a través d'un *dinamòmetre digital (Microfeet 2)* i l'explosiva s'avaluarà a través del salt, observant la força muscular concèntrica, durant la propulsió, i de manera excèntrica, per la recepció, a través del *"My jump".* Aplicació mòbil que mostra alta reproductivitat i fiabilitat en salts verticals, en comparació amb plataformes de força. (10).

Gràcies a la millora de lliscament entre els diferents plans tissulars que produeix la FD considerem interessant poder avaluar com es pot veure modificada la força en modalitats isomètriques, però també explosives, donat que ambdues presenten diferencies en el desplaçament de les fibres muscular durant la contracció. A més, estudis previs ja han demostrat millores en la força de grip sense dolor, per pacients amb epicodilalgia lateral crònica després de la FD (4).

- **Funció neuromuscular (FNM).** Perquè el múscul entri en activitat contràctil, ha de ser activat per les fibres nervioses motores, que provenen del sistema nerviós central, a les diferents zones d'innervació muscular.

  Electromiograma de superfície (EMGS). Eina no invasiva utilitzada per l'anàlisi de l'activitat muscular, que permet obtenir informació de la musculatura en repòs o moviment.

  Ha estat utilitzada com a eina d'avaluació en processos patològics però també com a suport en intervencions clíniques. Permet determinar si un múscul està involucrat en una determinada acció, i també detectar com es coordinen els músculs entre sí (11). De la mateixa manera, que amb la força, es considera d'interès conèixer l'activitat muscular durant el desenvolupament d'una activitat, tal i com permet avaluar la EMS. Millores sobre la funcionalitat han estat reportades en estudis clínics, tant en extremitat superior com inferior, després de l'aplicació de la tècnica (2)(4).

  Les variables de resposta fase 2:(taula 2).

TAULA 2. Variables de resposta estudiades.

**BACK SAVER SIT AND REACH**	**DINAMÒMETRE** (microfet 2)	**MY JUMP**	**EMGS** (Mdurance pro-motion)
Flexibilitat musculatura isquiosural (cm)	Força muscular isomètrica musculatura isquiosural i glútia (Kg)	Força muscular explosiva (Alçada vertical i pic de potència)	semitendinós, bíceps femoral i multífids (zona lumbar)<br><br>Màxima contracció voluntària (MCV)<br>Valor quadràtic mig (RMS)<br><br>Freqüència mitjana (FMED)<br><br>Velocitat de conducció (VC)<br><br>Zones d'innervació (ZI)

Procediment. Avaluador emmascarat i experimentat prendrà valors de les variables a estudiar.

Per l'EMGS, es col.locaràn els elèctrodes a la musculatura de la cadena posterior de l'extremitat inferior aleatoritzada.

Anàlisi estadístic. Es realitzarà amb el programa SPSS Statistics v.26.0. Es calcularan estadístics descriptius. Les variables qualitatives s'expressaran en número i percentatge, i les quantitatives i les seves diferències com a mitjana i desviació estàndard.

Es comprovarà la distribució normal de les variables quantitatives mitjançant el test de Kolmogorov-Smirnov (correccions de Lilliefors) o la prova de Shapiro-Wilk, segons la mida de la mostra. Es comprovarà l'homogenietat de les dades entre extremitats a l'inici. A les variables qualitatives s'utilitzarà la Chi quadrat o l'estadístic exacte de Fisher en cas contrari. Per les variables quantitatives s'utilitzarà la t Student o la prova de Wilcoxon segons els resultats de la prova de normalitat.

Es comprovaran l'homocedasticitat amb el test de Levene o la esfericitat segons la proba de Mauchly.

Es realitzaran un analisi intragrup mitjançant la prova ANOVA de mostres repetides amb la prova pos hoc de Bonferroni i, en cas de no cumplirse la normalitat, el test de Friedman amb la comparació per parells de Wilcoxon. En la comparació entre grups, es realitzará una comparació de les diferencies entre els diferentes periodes i s'analitzarà mitjançant la prova t Student o la prova de

Wilcoxon. En el cas de les variables qualitatives en la comparació entre grups s'utilitzarà la Chi cuadrat o l'estadistic exacte de Fisher. $p<0.05$ amb un 95% d'interval de confiança.

Aspectesètics.

- Aprovació prèvia del projecte per part del Comitè d'Ètica de Recerca (CER) UIC-Barcelona
- Es respectaran les directrius de la declaració de Helsinki (1973).
- Es sol·licitarà firma del consentiment informat i es respectarà la Llei orgànica 3/2018, de 5 de desembre, de Protecció de Dades de Caràcter Personal.

4. Pla de treball (cronograma)

	2019											
	Semestre I						Semestre II					
FASES	GENER	FEBRER	MARÇ	ABRIL	MAIG	JUNY	JULIOL	AGOST	SETEMBRE	OCTUBRE	NOVEMBRE	DESEMBRE
I. Plà de recerca												
Cerca i revisió bibliogràfica									■	■	■	■
Plantejament del problema									■	■	■	■
Redacció projecte									■	■	■	■
Enviament al director												
II. Comissió Acadèmica Doctorat												
Enviament projecte CAD												
Presentació CED												
Modificacions CED												
III. Marc teòric												
Redacció Marc teòric/Metodologia												
IV. Desenvolupament investigació												
Enviament projecte CER												
Entrenament procediment												
Reclutament de la mostra												
Recollida de dades												
Anàlisi de dades												
Redacció resiultats												
Correccions												
V. Conclusions												
Redacció de conclusions												
Correccions												
VI. Tràmits lectura tesi doctoral												
Enviar esborrany tesi al director												
Correccions												
Diposit tesi												
Tàmits administratius defensa tesi												
Defensa tesi												

	2020											
	Semestre I						Semestre II					
FASES	GENER	FEBRER	MARÇ	ABRIL	MAIG	JUNY	JULIOL	AGOST	SETEMBRE	OCTUBRE	NOVEMBRE	DESEMBRE
I. Plà de recerca												
Cerca i revisió bibliogràfica												
Plantejament del problema												
Redacció projecte	■	■										
Enviament al director	■	■										
II. Comissió Acadèmica Doctorat												
Enviament projecte CAD		■	■									
Presentació CED			■									
Modificacions CED				■								
III. Marc teòric												
Redacció Marc teòric/Metodologia				■	■	■	■	■	■	■	■	■
IV. Desenvolupament investigació												
Enviament projecte CER				■	■							
Entrenament procediment				■	■	■	■					
Reclutament de la mostra									■	■	■	■
Recollida de dades									■	■	■	■
Anàlisi de dades												
Redacció resiultats												
Correccions												
V. Conclusions												
Redacció de conclusions												
Correccions												
VI. Tràmits lectura tesi doctoral												
Enviar esborrany tesi al director												
Correccions												
Diposit tesi												
Tàmits administratius defensa tesi												
Defensa tesi												

	2021											
	Semestre I						Semestre II					
FASES	GENER	FEBRER	MARÇ	ABRIL	MAIG	JUNY	JULIOL	AGOST	SETEMBRE	OCTUBRE	NOVEMBRE	DESEMBRE
I. Plà de recerca												
Cerca i revisió bibliogràfica												
Plantejament del problema												
Redacció projecte												
Enviament al director												
II. Comissió Acadèmica Doctorat												
Enviament projecte CAD												
Presentació CED												
Modificacions CED												
III. Marc teòric												
Redacció Marc teòric/Metodologia	■	■	■	■	■	■	■		■	■	■	■
IV. Desenvolupament investigació												
Enviament projecte CER												
Entrenament procediment												
Reclutament de la mostra												
Recollida de dades	■	■	■	■	■	■	■		■	■	■	■
Anàlisi de dades												
Redacció resiultats												
Correccions												
V. Conclusions												
Redacció de conclusions												
Correccions												
VI. Tràmits lectura tesi doctoral												
Enviar esborrany tesi al director												
Correccions												
Diposit tesi												
Tàmits administratius defensa tesi												
Defensa tesi												

	2022											
	Semestre I						Semestre II					
FASES	GENER	FEBRER	MARÇ	ABRIL	MAIG	JUNY	JULIOL	AGOST	SETEMBRE	OCTUBRE	NOVEMBRE	DESEMBRE
I. Plà de recerca												
Cerca i revisió bibliogràfica												
Plantejament del problema												
Redacció projecte												
Enviament al director												
II. Comissió Acadèmica Doctorat												
Enviament projecte CAD												
Presentació CED												
Modificacions CED												
III. Marc teòric												
Redacció Marc teòric/Metodologia												
IV. Desenvolupament investigació												
Enviament projecte CER												
Entrenament procediment												
Reclutament de la mostra												
Recollida de dades												
Anàlisi de dades	■	■	■									
Redacció resiultats				■	■	■						
Correccions					■	■						
V. Conclusions												
Redacció de conclusions						■	■					
Correccions						■	■					
VI. Tràmits lectura tesi doctoral												
Enviar esborrany tesi al director							■		■			
Correccions										■		
Diposit tesi										■	■	
Tàmits administratius defensa tesi										■	■	
Defensa tesi												■

5. **Limitacions**

- La resposta neuromuscular es realitzi sobre subjectes sense patologia.

- L'especicficitat i concreció de les característiques de la mostra no representa la totalitat de la població diana suceptible a rebre tractament amb FD.

- Avaluació a curt termini, després d'una sola sessió.

6. **Experiència dels directors de tesi en l'àrea de coneixement.**

Dr. Carlos López de Celis: **Doctor per la Universidad de Zaragoza** (2016), **Máster en Investigación en Atención Primaria**, per la **UMH** i per la **UAB** (2009-2012).

Professor **Lector** de la UIC Barcelona, y Fisioterapeuta titular del ICS.

Investigador principal del Grupo de Estudio en Patología del Aparato Locomotor en Atención Primaria (GEPALAP) acreditado per IDIAP Jordi Gol.

- **Effectiveness of Diacutaneous Fibrolysis for the treatment of chronic Lateral Epicondylalgia. A randomized clinical trial.** López de Celis C, Barra López ME, González Rueda V, Bueno Gracia E, Rodríguez Rubio PR, Tricás Moreno JM. Clin Rehabil. 2018;32(5):644–653. (**Q1**)
- **Effectiveness of Diacutaneous Fibrolysis for the treatment of subacromial impingement syndrome: A randomised controlled trial.** Barra López ME, López de Celis C, Fernández Jentsch G, Raya de Cárdenas L, Lucha López MO, Tricás Moreno JM. Manual Therapy. 2013;18(5);418-424. (**Q2**)
- **The immediate effects of diacutaneous fibrolysis on pain and mobility in patients suffering from painful shoulder: a randomized placebo-controlled pilot study.** Barra López ME, López de Celis C, Fernández Jentsch G, Murilo Barrios E, Villar Mateo E, Raya Cardenas L. Clinical Rehabilitacion. 2011;25:339-348. (**Q1**)

Dr. Pere Ramón Rodriguez Rubio: **Doctor en Fisioteràpia** UIC-Barcelona (2014). **Màster Universitari en Fisioteràpia i Evidència Científica**. (2012)

Professor **Adjunt i sub-director d**el Departament de Fisioterapia de la UIC-Barcelona

- **Effectiveness of Diacutaneous Fibrolysis for the treatment of chronic Lateral Epicondylalgia. A randomized clinical trial.** López de Celis C, Barra López ME, González

Rueda V, Bueno Gracia E, Rodríguez Rubio PR, Tricás Moreno JM. Clin Rehabil. 2018;32(5):644–653. (**Q1**)

7. Finançament i conflicte d'interès.

Es preveu presentarse a beques competitives durante el proces.

8. Bibliografia mes rellevant.

1.- Barra ME, López C, Fernández G, Murillo E, Villar E, Raya L. The immediate effects of diacutaneous fibrolysis on pain and mobility in patients suffering from painful shoulder: A randomized placebo-controlled pilot study. Clin Rehabil. 2011;25(4):339–48.

2.- Fanlo-Mazas P, Bueno-Gracia E, de Escudero-Zapico AR, Tricás-Moreno JM, Lucha-López MO. The Effect of Diacutaneous Fibrolysis on Patellar Position Measured Using

Ultrasound Scanning in Patients With Patellofemoral Pain Syndrome. J Sport Rehabil. 2018;28(6):564–9.

3.- Jiménez Del Barrio S, Estébanez de Miguel E, Bueno Gracia E, Haddad Garay M, Tricás Moreno JM, Hidalgo García C. Effects of diacutaneous fibrolysis in patients with mild to moderate symptomatic carpal tunnel syndrome: a randomized controlled trial. Clin Rehabil. 2018;32(12):1645-1655.

4.- López-de-Celis C, Barra-López ME, González-Rueda V, Bueno-Gracia E, Rodríguez-Rubio PR, Tricás-Moreno JM. Effectiveness of diacutaneous fibrolysis for the treatment of chronic lateral epicondylalgia: a randomized clinical trial. Clin Rehabil. 2018;32(5):644–53.

5.- Martín-Rodríguez S, Loturco I, Hunter AM, Rodríguez-Ruiz D, Munguia-Izquierdo D. Reliability and Measurement Error of Tensiomyography to Assess Mechanical Muscle Function: A Systematic Review. J Strength Cond Res. 2017;31(12):3524-3536.

6.- Lohr C, Schmidt T, Medina-Porqueres I, Braumann KM, Reer R, Porthun J. Diagnostic accuracy, validity, and reliability of Tensiomyography to assess muscle function and exercise-induced fatigue in healthy participants. A systematic review with meta-analysis. J Electromyogr Kinesiol. 2019;47:65-87.

7.- Dellalana LE, Chen F, Vain A, Gandelman JS, Põldemaa M, Chen H, et al. Reproducibility of the durometer and myoton devices for skin stiffness measurement in healthy subjects. Ski Res Technol. 2019;25(3):289–93.

8.- Hven L, Frost P, Bonde JP. Evaluation of Pressure Pain Threshold as a Measure of Perceived Stress and High Job Strain. PLoS One. 2017;12(1):e0167257.

9.- Hui SSC, Yuen PY. Validity of the modified back-saver sit-and-reach test: A comparison with other protocols. Med Sci Sports Exerc. 2000;32(9):1655–9.

10.- Cruvinel-Cabral RM, Oliveira-Silva I, Medeiros AR, Claudino JG, Jiménez-Reyes P, Boullosa DA. The validity and reliability of the "My Jump App" for measuring jump height of the elderly. PeerJ. 2018;6:e5804.

11.- Bussey MD, Aldabe D, Adhia D, Mani R. Reliability of surface electromyography activity of gluteal and hamstring muscles during sub-maximal and maximal voluntary isometric contractions. Musculoskelet Sci Pract. 2018;34:103-107.

12.- Tricás JM, Lucha O, Duby P. Fibrolisis Diacutánea según el concepto de Kurt Ekman. Zaragoza: Asociación Española de Fibrolisis Diacutánea; 2010.

13. da Silva Dias R, Gómez-Conesa A. Síndrome de los isquiotibiales acortados. Fisioterapia. 2008;30(4):186–93.

14.- Butler D. The sensitive nervous system. Noigroup Publications. 2006

15.- Leandro Hernán Caamaño Barrios. Directores. Dr. Ricardo Ortega Santiago Dr. Fernando Galán del Río. Evaluación de las alteraciones de la mecanosensibilidad, puntos gatillo miofasciales y movilidad neural en mujeres con cefalea tensional frecuente episódica. Unversidad Rey Juan Carlos. Madrid. 2020.

16.- Chen J, Choi MSE, Kim MSM. Immediate Effect of Intermittent Versus Continuous Hamstring Static Stretching on the Muscle Tone and Range of Motion. J Korean Soc Phys Med 2019;14(4):19-27
